# Identification of Terfenadine as an Inhibitor of HumanCD81-Receptor HCV-E2 Interaction: Synthesis and Structure Optimization

**DOI:** 10.3390/molecules13051081

**Published:** 2008-05-07

**Authors:** Marcel Holzer, Sigrid Ziegler, Beatrice Albrecht, Bernd Kronenberger, Artur Kaul, Ralf Bartenschlager, Lars Kattner, Christian D. Klein, Rolf W. Hartmann

**Affiliations:** 1 Pharmaceutical and Medicinal Chemistry, Saarland University, PO Box 151150, D-66041 Saarbrücken, Germany; 2 Department of Molecular Virology, University of Heidelberg, Im Neuenheimer Feld 345, D-69120 Heidelberg, Germany; 3 Endotherm GmbH, Science-Park II, D-66123 Saarbrücken, Germany

**Keywords:** Hepatitis C Virus, CD81-receptor, large extracellular loop, terfenadine derivatives, microwave assisted syntheses

## Abstract

Terfenadine (4-[4-(hydroxydiphenylmethyl)-1-piperidyl]-1-(4-*tert*-butyl-phenyl)-butan-1-ol) was identified in a biological screening to be a moderate inhibitor (27 % inhibition) of the CD81-LEL–HCV-E2 interaction. To increase the observed biological activity, 63 terfenadine derivates were synthesized via microwave assisted nucleophilic substitution. The prepared compounds were tested for their inhibitory potency by means of a fluorescence labeled antibody assay using HUH7.5 cells. Distinct structure-activity relationships could be derived. Optimization was successful, leading to **3g**, identfied as the most potent compound (69 % inhibition). Experiments with viral particles revealed that there might be additional HCV infection reducing mechanisms.

## Introduction

Despite the discovery of the Hepatitis C Virus (HCV) more than 15 years ago, chronic HCV infection is still incurable in many patients, leading to cirrhosis, end-stage liver disease and hepatocellular carcinoma [1]. According to the 2002 WHO report, in 2001 more than 280,000 deaths worldwide were attributable to HCV infection [2]. The *large extracellular loop* (LEL) of the human CD81 cell surface protein, a member of the tetraspanin family, was identified as a binding partner for the Hepatitis C Virus envelope glycoprotein E2 (HCV-E2) [3]. Since inhibition of this interaction prevents HCV from infecting hepatocytes, the HCV principal target cells [3], the aim of the present work was to prepare compounds which restrain the CD81-LEL–HCV-E2 interaction by binding to the LEL. Another approach to inhibit this interaction using compounds that bind to the E2 glycoprotein of HCV was recently published by Van Compernolle *et al*. [3]. 

## Results and Discussion

### Biological Screening

The starting point of this work was a biological screening of natural products, current drugs and our in-house substance library (approximately 350 compounds, including several structurally different antihistamines) using a medium throughput assay developed in our group [4]. This assay is based on a procedure developed by Pileri *et al*. [5], in which the compounds inhibit the binding of the fluorescence-labeled CD81 antibody JS81 to HUH7.5 cells.

**Figure 1:**
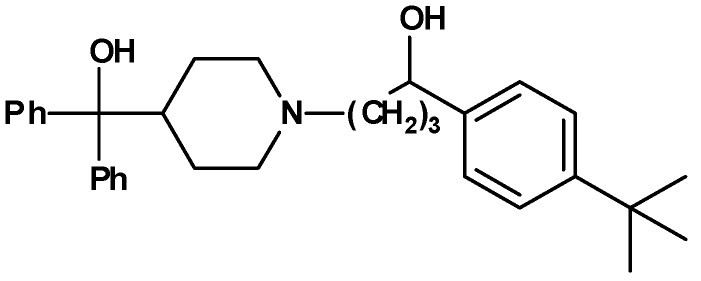
Terfenadine.

As an outcome of this screening an antihistamine,, terfenadine (Figure 1), was found to be a moderate inhibitor of the CD81-LEL–HCV-E2 interaction (27 % at 50 µM), whereas all other antihistamines showed no biological activity.

### Structure Modification

Based on these results, a series of terfenadine derivatives was prepared seeking to increase the inhibitory activity and to derive structure-activity relationships. The following structural features were modified: length of the alkyl “linker” between the piperidine and the phenyl moiety, alkyl substituent on the phenyl ring, secondary hydroxy group and the azacyclonol moiety.

### Syntheses

**Scheme 1:**
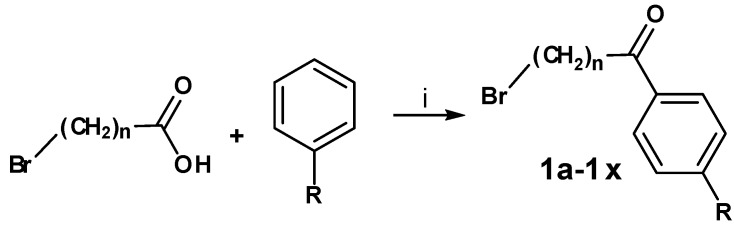
Reagents and conditions for the FC acylation and synthesized 1-aryl-ω-bromo ketones.

The first step of the preparation was a Friedel-Crafts (FC) acylation of the benzene derivative with aluminium chloride as catalyst and the carboxylic acid chloride of the corresponding ω-bromo-carboxylic acid, prepared using thionyl chloride (Scheme 1). The synthesized 1-aryl-ω-bromo ketones are shown in Table 1.

*Reagents and conditions*: (i) 1. SOCl_2_, rt, 30 min 2. dry CH_2_Cl_2_, AlCl_3_, 0 °C, 45 min (n = 3-5; R = H; C_1_-C_4_).

**Table 1:** Synthesized 1-aryl-ω-bromo ketones **1a-1x** (n = 3-5; R = H; C_1_-C_4_).

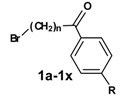						
**R**	**n**	**compound**	**n**	**compound**	**n**	**compound**
H	5	**1a**	4	**1i**	3	**1q**
Methyl	5	**1b**	4	**1j**	3	**1r**
Ethyl	5	**1c**	4	**1k**	3	**1s**
*n*-Propyl	5	**1d**	4	**1l**	3	**1t**
*iso*-Propyl	5	**1e**	4	**1m**	3	**1u**
*n*-Butyl	5	**1f**	4	**1n**	3	**1v**
*iso*-Butyl	5	**1g**	4	**1o**	3	**1w**
*tert*-Butyl	5	**1h**	4	**1p**	3	**1x**

**Scheme 2:**
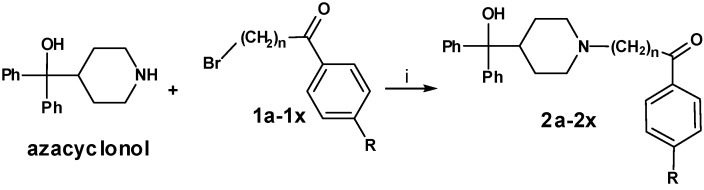
*Reagents and conditions*: (i) K_2_CO_3_, catalytic KI and 18-crown-6, dry acetonitrile, microwave: 150 Watt, 6.5 bar, 175 °C, 45 min (100 Watt, 140 °C, 3 h: **2g**, **2h**, **2t**, **2u**, **2v**, **2w**), n = 3-5; R = H; C_1_-C_4_.

In the second reaction step (Scheme 2) the precursors **1a-1x** were coupled to azacyclonol according to a described procedure [6]. The classical nucleophilic substitution was optimized for microwave assisted synthesis. The desired compounds were obtained in satisfying yields in a very short time (5-45 minutes). This led to compounds **2a-2x**, shown in Table 2.

**Table 2:** Synthesized terfenadine derivatives **2a-2x** and **3a-3w** (n = 3-5; R = H; C_1_-C_4_).

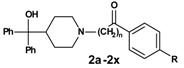							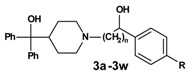						
**R**	**n**	**compound**	**n**	**compound**	**n**	**compound**	**n**	**compound**	**n**	**compound**	**n**	**compound**
H	5	**2a**	4	**2i**	3	**2q**	5	**3a**	4	**3i**	3	**3q**
Methyl	5	**2b**	4	**2j**	3	**2r**	5	**3b**	4	**3j**	3	**3r**
Ethyl	5	**2c**	4	**2k**	3	**2s**	5	**3c**	4	**3k**	3	**3s**
*n*-Propyl	5	**2d**	4	**2l**	3	**2t**	5	**3d**	4	**3l**	3	**3t**
*iso*-Propyl	5	**2e**	4	**2m**	3	**2u**	5	**3e**	4	**3m**	3	**3u**
*n*-Butyl	5	**2f**	4	**2n**	3	**2v**	5	**3f**	4	**3n**	3	**3v**
*iso*-Butyl	5	**2g**	4	**2o**	3	**2w**	5	**3g**	4	**3o**	3	**3w**
*tert*-Butyl	5	**2h**	4	**2p**	3	**2x**	5	**3h**	4	**3p**	3	**terfenadine**

**Scheme 3:**
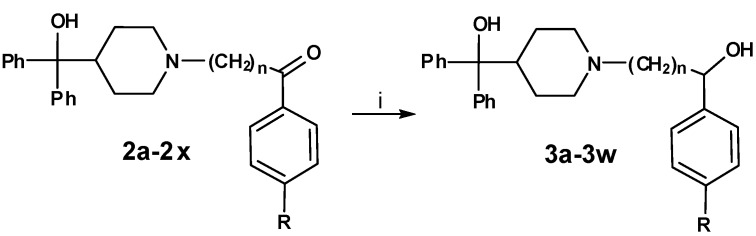
*Reagents and conditions*: (i) NaBH_4_, MeOH, 0 °C, 1 h (n = 3-5; R = H; C_1_-C_4_).

A sodium borohydride reduction (Scheme 3) of the ketone function followed. The prepared alcohols **3a-3w**, which were obtained as racemates, are also listed in Table 2.

Since azacyclonol, the second component for the coupling reaction, was commercially available, this synthetic procedure facilitated the preparation of a large variety of compounds in a minimum amount of time. For further modification of the secondary hydroxy group the ester **5a**, the amide **5b** and the alkane **8a** were synthesized. 

**Scheme 4:**
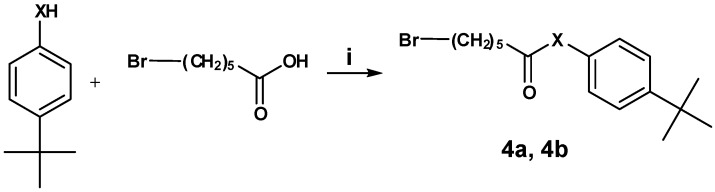
*Reagents and conditions*: (i) 1. SOCl_2_, rt, 1 h 2. NEt_3_, dry CH_2_Cl_2_, 0 °C 30 min, rt 1 h (X = -O-: **4a**, X = -NH-: **4b**).

Activation of 6-bromo hexanoic acid with thionyl chloride followed by addition of commercially available 4-*tert*-butyl phenol and 4-*tert*-butyl aniline, respectively, led to the ω-bromo substituted precursors **4a** and **4b** (Scheme 4).

**Scheme 5:**
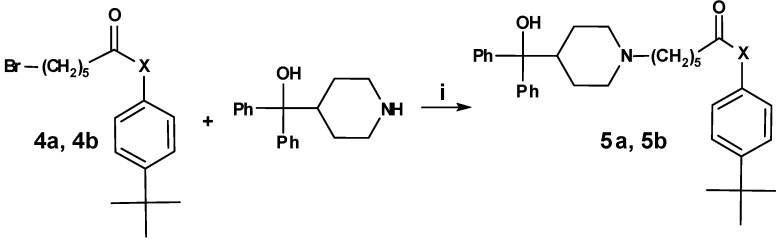
*Reagents and conditions*: (i) K_2_CO_3_, catalytic KI and 18-crown-6, dry acetonitrile, 150 Watt, 140 °C, 1 h (X = -O-: **5a**, X = -NH-: **5b**).

Next, these precursors were nucleophilically coupled to azacyclonol, leading to compounds 5a and 5b, respectively (Scheme 5).

**Scheme 6:**
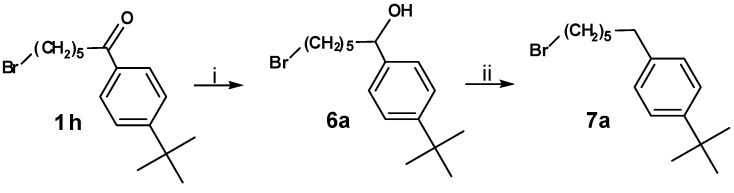
*Reagents and conditions*: (i) see Scheme 3; (ii) indium(III) chloride, chlorodiphenylsilane, dry dichloromethane, 3 h, rt.

**Scheme 7:**
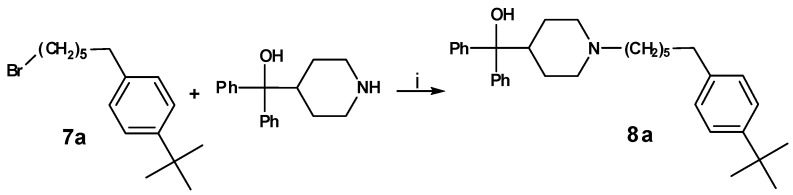
*Reagents and conditions*: (i) K_2_CO_3_, catalytic KI and 18-crown-6, dry acetonitrile, 50 Watt, 100 °C, 5 min

For the synthesis of **8a **compound **1h** was reduced using sodium borohydride. The resulting alcohol **6a** was converted into the corresponding alkane **7a ** by means of a mixture of indium(III) chloride and chlorodiphenylsilane (Scheme 6) and subsequently coupled to azacyclonol, leading to compound **8a** (Scheme 7).

**Scheme 8:**
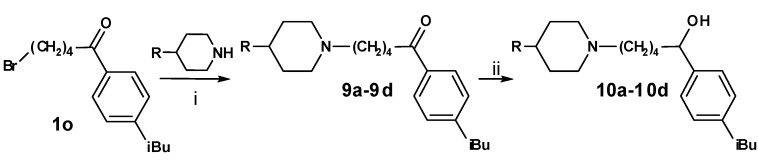
*Reagents and conditions*: (i) K_2_CO_3_, catalytic KI and 18-crown-6, dry acetonitrile, microwave 150 Watt, 6.5 bar, 175 °C, 45 min; (ii) NaBH_4_, MeOH, 0 °C, 1 h (R = -H, -CH_3_, -OH, benzyl).

The exchange of the azacyclonol component by smaller piperidine moieties started from the 1-aryl-ω-bromo ketone **1o ** and was accomplished via microwave assisted nucleophilic substitution, followed by reduction of the ketone (Scheme 8). The prepared compounds **9a**-**9d**, as well as the corresponding alcohols **10a-10d**, obtained as racemates, are shown in Table 3.

**Table 3:** Synthesized terfenadine derivatives **9a-9d** and **10a-10d**.

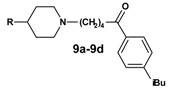		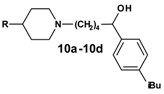	
**R**	**compound**	**R**	**compound**
H	**9a**	H	**10a**
Methyl	**9b**	Methyl	**10b**
Hydroxyl	**9c**	Hydroxyl	**10c**
Benzyl	**9d**	Benzyl	**10d**

**Scheme 9:**
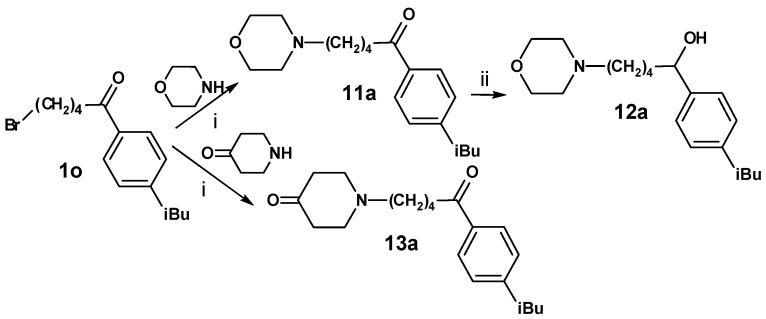
*Reagents and conditions*: (i) K_2_CO_3_, catalytic KI and 18-crown-6, dry acetonitrile, microwave 150 Watt, 6.5 bar, 175 °C, 45 min; (ii) NaBH_4_, MeOH, 0 °C, 1 h.

Furthermore, **1o** was reacted with morpholine and piperidin-4-one resulting in compounds **11a**, **12a** and **13a** (scheme 9). The morpholine derivative **12a** was obtained as a racemate. 

**Scheme 10:**
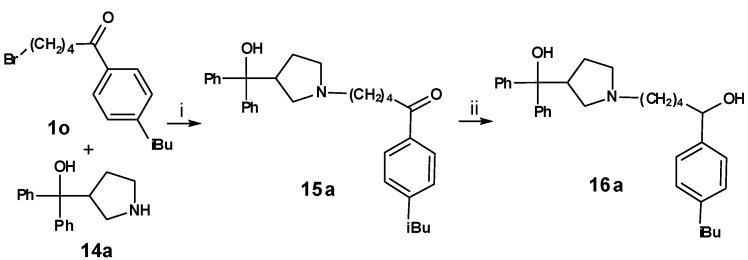
*Reagents and conditions*: (i) K_2_CO_3_, catalytic KI and 18-crown-6, dry acetonitrile, microwave 150 Watt, 6.5 bar, 175 °C, 45 min; (ii) NaBH_4_, MeOH, 0 °C, 1 h.

For the syntheses of the pyrrolidine compounds **15a** and **16a**, **1o** was coupled with diphenyl(pyrrolidin-3-yl)methanol (**14a**). The resulting ketone **15a** was reduced with sodium borohydride (Scheme 10), leading to the desired compound as a racemate.

### Biological evaluation

The synthesized terfenadine derivatives were tested for their inhibitory potency using the antibody neutralization (AN) assay mentioned above (Table 4, Table 5, Table 6, Table 7 and Table 8) [4]. In this assay the potential inhibitors and the fluorescence-labeled antibody JS81 compete for binding to the LEL on the CD81 receptor molecule. The reduction of the interaction of JS81 with CD81 on HUH7.5 cells caused by the compounds decreases fluorescence which is measured by FACS in comparison to untreated control cells.

The % inhibition values of compounds **2a-2x** (Table 4) and **3a-3w** (Table 5) show that the alkyl substituent R on the phenyl ring has a major influence on the activity. Another important feature is obviously the length of the linker, whereas the activities of the ketones (Table 4) and the corresponding alcohols (Table 5) do not differ significantly, at least in case of the *n*-propyl and *n*-butyl compounds. 

**Table 4:** Inhibition of protein interaction in AN assay by compounds **2a**-**2x **(concentration: 50 µM, standard deviation ≤ 6 %).

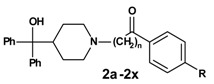						
	**n = 5**		**n = 4**		**n = 3**	
**R**	**compound**	**inhibition (%)**	**compound**	**inhibition (%)**	**compound**	**inhibition (%)**
H	**2a**	10	**2i**	4	**2q**	7
Methyl	**2b**	12	**2j**	8	**2r**	7
Ethyl	**2c**	42	**2k**	24	**2s**	1
*n*-Propyl	**2d**	59	**2l**	64	**2t**	57
*iso*-Propyl	**2e**	63	**2m**	29	**2u**	41
*n*-Butyl	**2f**	56	**2n**	57	**2v**	56
*iso*-Butyl	**2g**	46	**2o**	60	**2w**	44
*tert*-Butyl	**2h**	49	**2p**	53	**2x**	37

**Table 5:** Inhibition of protein interaction in AN assay by compounds **3a-3w** (concentration: 50 µM, standard deviation ≤ 6 %).

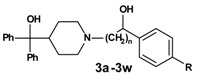						
	**n = 5**		**n = 4**		**n = 3**	
**R**	**compound**	**inhibition (%)**	**compound**	**inhibition (%)**	**compound**	**inhibition (%)**
H	**3a**	4	**3i**	1	**3q**	5
Methyl	**3b**	8	**3j**	5	**3r**	4
Ethyl	**3c**	13	**3k**	4	**3s**	4
*n*-Propyl	**3d**	32	**3l**	53	**3t**	23
*iso*-Propyl	**3e**	9	**3m**	4	**3u**	11
*n*-Butyl	**3f**	49	**3n**	49	**3v**	64
*iso*-Butyl	**3g**	69	**3o**	67	**3w**	42
*tert*-Butyl	**3h**	58	**3p**	44	**terfenadine**	27

Increasing the size of R from hydrogen to bulkier alkyl substituents increases inhibition. A maximum inhibition, not depending on the linker length, could be reached by the use of *n*-propyl for the ketones and *iso*-butyl for the alcohols, with the exception of compounds **2d** and **3w**, which are less active than the analogous **2e **and **3v**. Generally, compounds with n = 3 show a lower inhibition than those with n = 4 or 5, with the exception of **3v**, which is more active than **3f **and **3n**. Furthermore, reduction of the ketones **2a**-**2x ** to the corresponding alcohols **3a**-**3w ** and terfenadine led to a decreased activity for compounds with small substituents at the phenyl ring, whereas a reduction of bulky substituted compounds led to derivatives with comparable inhibition. The reduction of the ketones with R = *iso*-propyl to the corresponding alcohols led to a nearly complete loss of biological activity. The most active compound in this series of terfenadine derivatives is **3g **(69 % inhibition). Therefore, the hexanol linker combined with an *iso*-butyl group at the phenyl ring is the most favorable substitution pattern in this class of compounds.

Replacement of the azacyclonol moiety by smaller piperidine residues (**9a**-**9c**, **10a**-**10c** and **11a**-**13a**) led to a loss of inhibitory activity compared to the reference compounds **2o** and **3o **(Table 6). Substitution of the piperidine group by a 4-benzyl group (**9d** and **10d**) increased the inhibition to a moderate level. Again bulky substituents are essential for activity at this part of the molecule.

**Table 6:** Inhibition of protein interaction in AN assay by compounds **9a-13a** compared to compounds **2o** and **3o** (concentration: 50 µM, standard deviation ≤ 6 %).

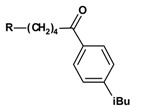			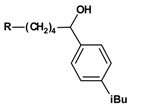	
**R**	**compound**	**inhibition (%)**	**compound**	**inhibition (%)**
azacyclonol	**2o**	60	**3o**	67
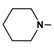	**9a**	7	**10a**	5
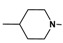	**9b**	5	**10b**	7
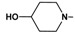	**9c**	7	**10c**	6
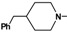	**9d**	29	**10d**	31
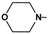	**11a**	8	**12a**	4
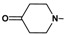	**13a**	5		

Reduction of the secondary hydroxy group of **3h** to the corresponding alkane **8a** led to a strong decrease of inhibition (Table 7). Exchange of the ketone of **2h** by an ester function (compound **5a**) did not influence the inhibitory activity, whereas an amide group (compound **5b**) increased the inhibition. These SAR results indicate that the functional group X might act as an H-bond acceptor interacting with the CD81 protein.

**Table 7:** Inhibition of protein interaction in AN assay by compounds **5a**-**5b**, **8a** compared to compounds **2h** and **3h **(R = azacyclonol, concentration: 50 µM, standard deviation ≤ 6 %).

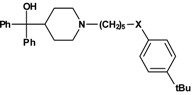					
**X**	**compound**	**inhibition (%)**	**X**	**compound**	**inhibition (%)**
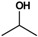	**3h**	58	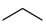	**8a**	9
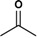	**2h**	49	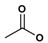	**5a**	48
			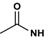	**5b**	57

Exchange of the piperidine of the azacyclonol moiety of **2o** and **3o** by pyrrolidine (compounds **15a** and **16a**) did not lead to a significant change of the inhibitory activity (Table 8). Obviously due to the flexibility of the chain, appropriate conformations can be found for both heterocycles.

**Table 8:** Inhibition of protein interaction in AN assay by compounds 15a, 16a compared to compounds 2p and 3p (R = azacyclonol, concentration: 50 µM, standard deviation ≤ 6 %).

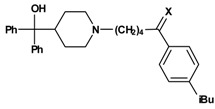			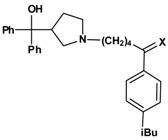	
**X**	**compound**	**inhibition (%)**	**compound**	**inhibition (%)**
O	**2o**	60	**15a**	64
OH	**3o**	67	**16a**	62

Selected compounds with high, moderate and low inhibition in the AN assay were tested for their inhibition in an infectivity assay developed by Pietschmann and coworkers [7] (Table 9), in which HUH7.5 cells were incubated with HCV particles of the LUC-Jc1 genome (genotype 2a) [8] and potential inhibitors. After 48 hours, the cells were lysed and luciferase activity (a marker of infection and viral replication) was measured. The infectivity assay was performed at the highest non-cytotoxic concentrations of the compounds (0.5 µM: **2e**, **2s**, **3f**, **3g**, **3i**, **8a** and 5 µM: **3q**, **10d**).

Compounds with high biological activity in the neutralization assay showed good inhibition in the infectivity assay as well. This clearly indicates that inhibition of the protein-protein interaction leads to a reduction of infectivity. On the other hand there are compounds which reduced infectivity without having been active in the AN assay. One plausible reason for this phenomenon could be an interaction of these compounds with an additional target which is involved in viral infection. 

**Table 9:** Inhibition of infectivity of viral particles by selected compounds.

Compound	**Antibody neutralization assay (inhibition (%), 50 µM)**	Infectivity assay with viral particles **(inhibition (%), 0.5 µM)**
**2e**	63	43
**2s**	1	19
**3f**	49	40
**3g**	69	32
**3i**	1	27
**3q**	5	33^a^
**8a**	9	27
**10d**	31	45^a^

Concentration: 0.5 µM, ^a ^5 µM, standard deviation ≤ 14 % compared to AN assay data.

## Conclusions

After having identified terfenadine as an active compound inhibiting the CD81-LEL–HCV-E2 interaction by 27 %, the compound was structurally modified with the aim of increasing the activity. The results obtained clearly demonstrate that a bulky substituent at the phenyl moiety as well as an additional bulky substituent at the other part of the molecule (like azacyclonol) are necessary for activity as well as the secondary OH-group. Another H-bond acceptor like ketone, ester or amide can replace the latter. Importantly, the activity of the parent compound can be increased by elongation of the alkyl chain resulting in **3g** with an inhibition of 69 %. A further experiment with viral particles and HUH7.5 cells showed that there are possibly further mechanisms by which the test compounds unfold their anti-viral activity. These mechanisms remain to be elucidated.

## Experimental

### General

Solvents and reagents were used as received from commercial distributors without further purification. Anhydrous reactions were conducted under a nitrogen atmosphere. Proton and carbon NMR spectra (in CDCl_3_, unless otherwise noted) were recorded on a Bruker AM 500 instrument The proton NMR spectra were recorded at 500 MHz, the carbon NMR spectra at 125 MHz. Chemical shifts δ are reported in ppm units. Molecular mass was determined by liquid chromatography – tandem mass spectrometry (LC-MS/MS) using a TSQ Quantum from Thermo Finnigan equipped with an electro spray interface and connected to a Surveyor HPLC (Thermo Finnigan). Positive and negative ion mass spectra were recorded (mass range m/z 150–1500) in normal scan mode. Melting points were determined using a Stuart Scientific SMP3 melting point apparatus. IR measurements were performed on a Bruker Vector 33 at a frequency range from 4000–250 cm^-1^. Wave numbers υ are reported in cm^-1^. Microwave assisted syntheses was performed using a CEM DISCOVER microwave oven or a MLS MultiSYNTH microwave oven respectively. Preparative HPLC was carried out using an Agilent Technologies, 1200 Series Isocratic, with an Agilent Prep-C18 (5 µm; 30x100mm) preparative column using 70:30 methanol/water as eluent. Flash chromatography was performed using Merck silica gel 35/40–63/70.

### Antibody neutralization assay.

For the antibody neutralization assay HUH7.5 cells were used, derived from a human hepatoma cell line which was preselected via FACS for cells displaying high amounts of CD81 on their cell surface. Cells were grown under standard conditions (Dulbecco’s Modified Eagle’s Medium with 10 % FCS, 1% penicillin/streptavidin, pH 7.4, at 37°C with 5% CO_2_). 1∙10^5^ cells were incubated with 100 µL (50µM) of the potential inhibitor. The inhibitor solution contained 1 % DMSO, a concentration tolerated by the used HUH7.5 cells and without influence on MFI (mean fluorescence intensity) values (data not shown). Incubation was performed in 96 transwell plates for 10 minutes at room temperature. Subsequently 4 µL of the fluorescence-labeled anti human CD81 antibody JS81 (Pharmingen) and 21 µL of phosphate buffered saline buffer (PBS) were added and kept at room temperature for 10 minutes. The final concentration of JS81 in the assay was 1.6∙10^-3^ µg/µL. After addition of 125 µL of PBS the cell suspension was incubated in the dark for 5 hours followed by FACS (Becton Dickison – FACS Calibur; software: Cell Quest Pro) analysis. Ten thousand vital cells out of a predetermined area were analyzed for their MFI.

### Infectivity assay.

*In vitro* synthesis of the LUC-Jc1 RNA genome in which the luciferase reporter gene was inserted into the genome of the Jc1 HCV variant and electroporation of Huh-7 cells was performed as described previously [9, 10]. We collected culture medium containing viral particles 48 h after transfection. HUH7.5 target cells were seeded 24 h before infection at a density of 6∙10^5^ cells/well in a 24 well plate. Cells were infected with 200 µL inoculum containing the potential inhibitors at 5 µM or 0.5 µM for 4 h. Concentrations were chosen according to the highest non-cytotoxic concentration of the given compound. Cells were washed, complete medium was added and cells were cultured for 48 h. Afterwards cells were lysed for luciferase assay as previously described [7]. 

### Chemistry

#### *General procedure for* *the Friedel-Crafts-acylation*

The carboxylic acid was stirred with thionyl chloride (10 mL) at room temperature for 1h. After removing the excess of thionyl chloride under reduced pressure, dry dichloromethane (15 mL) and the benzene derivative (1 equivalent, referring to the carboxylic acid) were added. The solution was cooled to 0 °C and AlCl_3_ (1.2 equivalents) were added. After stirring at that temperature for 45 min, the mixture was hydrolyzed, the layers separated and the water phase was extracted with dichloromethane two times. The combined organic layers were dried and the solvent removed. Flash chromatography using 1/15 ethyl acetate/n-hexane as eluent led to the desired compounds.

#### *General procedure for the* *nucleophilic substitution*

Azacyclonol (1 equivalent), ketone (1 equivalent), potassium carbonate (5 equivalents), a catalytic amount of potassium iodide and 18-crown-6 were stirred in dry acetonitrile (4 mL) in the microwave (CEM Discover) at 150 Watt, 6.5 bar, 175 °C for 45 min or (MultiSYNTH) at 100 Watt, 140 °C, 3 h **(2g, 2h, 2t, 2u, 2v, 2w)** or at 50 Watt, 100 °C, 5 min (**8a**). The microwave assisted reactions were performed in a continuous air stream cooled closed vessel. Reaction temperature was monitored via an IR sensor in both microwave ovens and an additional fiber optic component in case of the MultiSYNTH. After filtering off the remaining precipitate the solvent was removed. The impurities could not be removed completely by flash chromatography (6/1 ethyl acetate/n-hexane + 3 % NEt_3_), so preparative HPLC (isocratic 70/30 MeOH/H_2_O) was performed and the pure product isolated.

#### *General procedure for the reduction of* *the ketones*

Sodium borohydride (5 equivalents) and ketone (1 equivalent) were added to 2 mL methanol at 0 °C. After stirring for 30 min a spatula tip of sodium borohydride was added to the mixture which was stirred for another 30 min at 0 °C. The mixture was hydrolyzed using 2 mL NH_4_Cl-solution. After removing the solvents under reduced pressure, the remaining solid was washed two times with methanol and ethyl acetate (2 mL each). The combined organic layers were freed from solvent and the raw product was purified by preparative HPLC (isocratic MeOH/H_2_O: 70/30) to give the racemates in satisfying yields.

#### *General procedure for the ester and amide* *coupling*

The carboxylic acid (1 equivalent) was stirred with an excess of thionyl chloride for 1 hour at room temperature. The resulting clear solution was freed from remaining thionyl chloride under reduced pressure. The acid chloride was dissolved in dry dichloromethane and added dropwise at 0 °C to a solution of the corresponding alcohol or amine (1.2 equivalents) and an equimolar amount of triethylamine in dry dichloromethane. After stirring for 30 minutes at 0 °C the mixture was warmed to room temperature and stirred for 1 hour. The precipitated solid was filtered off. The solvent was removed and the raw product purified by column flash chromatography using an ethyl acetate/n-hexane mixture.

*6-Bromo-1-phenylhexan-1-one* (**1a**). Yield: 26 %; ^1^H-NMR: 7.88–7.85 (2 H, m), 7.49–7.45 (1 H, m), 7.39–7.35 (2 H, m), 3.35–3.32 (2 H, m), 2.92–2.88 (2 H, m), 1.86–1.79 (2 H, m), 1.72–1.64 (2 H, m), 1.48–1.40 (2 H, m) [Lit. [11] 400 MHz, CDCl_3_: 7.94 (2 H, m), 7.55 (1 H, m), 7.45 (2 H, m), 3.41 (2 H, t, *J* = 6.80), 2.98 (2 H, t, *J* = 7.00), 1.90 (2 H, m), 1.76 (2 H, m), 1.53 (2 H, m)]; ^13^C-NMR: 199.96, 136.95, 133.02, 128.61, 128.02, 38.29, 33.69, 32.66, 27.89, 23.33.

*6-Bromo-1-(4-methylphenyl)hexan-1-one* (**1b**). Yield: 50 %; ^1^H-NMR: 7.76 (2 H, d, *J* = 8.06), 7.16 (2 H, d, *J* = 7.98), 3.34–3.31 (2 H, t, *J* = 6.78), 2.88–2.84 (2 H, t, *J* = 7.44), 2.31 (3 H, s), 1.85–1.78 (2 H, m), 1.70–1.62 (2 H, m), 1.47–1.39 (2 H, m) [Lit. [12] CDCl_3_: 7.85 (2 H, d, *J* = 7.30), 7.25 (2 H, d, *J* = 8.60), 3.42 (2 H, d, *J* = 8.60), 2.96 (2 H, t, *J* = 7.30), 2,41 (3 H, s), 1.97–1.86 (2 H, m), 1.82–1.74 (2 H, m), 1.58–1.47 (2 H, m)]; ^13^C-NMR: 199.61, 143.72, 134.47, 129.26, 128.13, 38.14, 33.67, 32.65, 27.89, 23.41, 21.62.

*6-Bromo-1-(4-ethylphenyl)hexan-1-one* (**1c**). Yield: 75 %; ^1^H-NMR: 7.89 (2 H, d, *J* = 8.34), 7.29 (2 H, d, *J* = 8.49), 3.43 (2 H, t, *J* = 6.79), 2.99–2.96 (2 H, m), 2.74–2.68 (2 H, m), 1.95–1.88 (2 H, m), 1.81–1.73 (2 H, m), 1.57–1.50 (2 H, m), 1.26 (3 H, t, *J* = 7.60) [Lit. [12] CDCl_3_: 7.88 (1 H, d, *J* = 8.60), 7.28 (1 H, d, *J* = 8.60), 3.43 (2 H, t, *J* = 7.00), 2.97 (2 H, t, *J* = 7.30), 2.71 (2 H, q, *J* = 7.20), 2.04–1.48 (6 H, m), 1.26 (3 H, t, *J* = 7.60)]; ^13^C-NMR: 199.65, 148.91, 133.65, 127.22, 127.06, 37.16, 32.67, 31.64, 27.91, 26.89, 22.41, 14.22.

*6-Bromo-1-(4-n-propylphenyl)hexan-1-one* (**1d**). Yield: 74 %; ^1^H-NMR: 7.84 (2 H, d, *J* = 8.32), 7.23 (2 H, d, *J* = 8.35), 3.40 (2 H, t, *J* = 6.78), 2.94 (2 H, t, *J* = 7.24), 2.63–2.60 (2 H, m), 1.92–1.86 (2 H, m), 1.77–1.71 (2 H, m), 1.67–1.60 (2 H, m), 1.53–1.47 (2 H, m), 0.92 (3 H, t, *J* = 7.34); ^13^C-NMR: 199.69, 148.45, 134.75, 128.71, 128.17, 38.20, 38.04, 33.69, 32.70, 27.95, 24.26, 23.46, 13.81.

*6-Bromo-1-(4-iso-propylphenyl)hexan-1-one* (**1e**). Yield: 65 %; ^1^H-NMR: 7.89 (2 H, d, *J* = 8.40), 7.30 (2 H, d, *J* = 8.14), 3.42 (2 H, t, *J* = 6.79), 2.99–2.93 (3 H, m), 1.94–1.88 (2 H, m), 1.79–1.73 (2 H, m), 1.56–1.49 (2 H, m), 1.26 (6 H, d, *J* = 6.93); ^13^C-NMR: 199.66, 154.50, 134.88, 128.31, 126.70, 38.21, 34.27, 33.69, 32.70, 27.96, 23.72. 23.47.

*6-Bromo-1-(4-n-butylphenyl)hexan-1-one* (**1f**). Yield: 44 %; ^1^H-NMR: 7.98 (2 H, d, *J* = 8.25), 7.37 (2 H, d, *J* = 8.03), 3.53 (2 H, t, *J* = 6.77), 3.09–3.06 (2 H, m), 2.79–2.76 (2 H, m), 2.05–2.00 (2 H, m), 1.91–1.85 (2 H, m), 1.76–1.70 (2 H, m), 1.67–1.61 (2 H, m), 1.51–1.43 (2 H, m), 1.04 (3 H, t, *J* = 7.32); ^13^C-NMR: 199.66, 148.69, 134.71, 128.66, 128.19, 38.20, 35.71, 33.68, 33.29, 32.70, 27.95, 23.46, 22.36, 13.95.

*6-Bromo-1-(4-iso-butylphenyl)hexan-1-one* (**1g**). Yield: 68 %; ^1^H-NMR: 7.89 (2 H, d, *J* = 8.28), 7.23 (2 H, d, *J* = 8.28), 3.43 (2 H, t, *J* = 6.79), 2.97 (2 H, t, *J* = 7.35), 2.21 (2 H, d, *J* = 7.21)*.* 1.95–1.88 (3 H, m), 1.81–1.75 (2 H, m), 1.57–1.51 (2 H, m), 0.92 (6 H, t, *J* = 6.62); ^13^C-NMR: 199.64, 147.44, 134.79, 129.31, 128.01, 45.38, 38.17, 33.61, 32.68, 30.11, 27.93, 23.43, 22.35.

*6-Bromo-1-(4-tert-butylphenyl)hexan-1-one* (**1h**). Yield: 60 %; ^1^H-NMR: 7.89 (2 H, d, *J* = 8.50), 7.47 (2 H, d, *J* = 8.52), 3.41 (2 H, t, *J* = 6.79), 2.96 (2 H, t, *J* = 7.35), 1.94–1.88 (2 H, m), 1.79–1.73 (2 H, m), 1.56–1.50 (2 H, m), 1.34 (9 H, s); ^13^C-NMR: 199.60, 156.68, 134.44, 128.00, 125.53, 38.18, 35.10, 33.62, 32.68, 31.12, 27.94, 23.46.

*5-Bromo-1-phenylpentan-1-one* (**1i**). Yield: 11 %; ^1^H-NMR: 7.96–7.94 (2 H, m), 7.58–7.54 (1 H, m), 7.48–7.45 (2 H, m), 3.38 (2 H, t, *J* = 6.62), 3.03–3.00 (2 H, m), 1.99–1.87 (4 H, m) [Lit. [13] CDCl_3_: 7.90–7.10 (5 H, m), 3.40 (2 H, t), 2.99 (2 H, t), 1.91 (4 H, m)]; ^13^C-NMR: 199.53, 136.80, 133.05, 128.59, 127.97, 37.38, 33.29, 32.16, 22.72.

*5-Bromo-1-(4-methylphenyl)pentan-1-one* (**1j**). Yield: 57 %; ^1^H-NMR: 7.83 (2 H, d, *J* = 8.20), 7.23 (2 H, d, *J* = 7.88), 3.43–3.41 (2 H, m), 2.97–2.94 (2 H, m), 2.38 (3 H, s), 1.94–1.84 (4 H, m); ^13^C-NMR: 199.16, 143.83, 134.38, 129.29, 128.13, 37.29, 33.41, 32.25, 22.86, 21.63.

*5-Bromo-1-(4-ethylphenyl)pentan-1-one* (**1k**). Yield: 68 %; ^1^H-NMR: 7.78 (2 H, d, *J* = 8.03), 7.17 (2 H, d, *J* = 8.03), 3.34–3.31 (2 H, m), 2.88–2.85 (2 H, m), 2.62–2.57 (2 H, m), 1.84–1.76 (4 H, m), 1.15 (3 H, t, *J* = 7.53); ^13^C-NMR: 199.19, 150.02, 134.66, 128.29, 128.15, 37.35, 33.39, 32.32, 28.98, 22.93, 15.25.

*5-Bromo-1-(4-n-propylphenyl)pentan-1-one* (**1l**). Yield: 55 %; ^1^H-NMR: 7.87 (2 H, d, *J* = 8.33), 7.26 (2 H, d, *J* = 8.45), 3.46–3.43 (2 H, m), 3.00–2.97 (2 H, m), 2.66–2.62 (2 H, m), 1.99–1.85 (4 H, m), 1.71–1.61 (2 H, m), 0.94 (3 H, t, *J* = 7.33); ^13^C-NMR: 199.26, 148.54, 134.63, 128.72, 128.15, 38.02, 37.32, 33.38, 32.27, 24.23, 22.88, 13.77.

*5-Bromo-1-(4-iso-propylphenyl)pentan-1-one* (**1m**). Yield: 33 %; ^1^H-NMR: 7.88 (2 H, d, *J* = 8.20), 7.30 (2 H, d, *J* = 8.20), 3.45 (2 H, t, *J* = 6.62), 2.99–2.93 (3 H, m), 1.98–1.85 (4 H, m), 1.26 (6 H, d, *J* = 6.94); ^13^C-NMR: 199.21, 154.57, 134.74, 128.28, 126.70, 37.31, 34.23, 33.38, 32.25, 23.67, 22.87.

*5-Bromo-1-(4-n-butylphenyl)pentan-1-one* (**1n**). Yield: 37 %; ^1^H-NMR: 7.78 (2 H, d, *J* = 8.33), 7.16 (2 H, d, *J* = 8.43), 3.36–3.33 (2 H, m), 2.90–2.87 (2 H, m), 2.59–2.55 (2 H, m), 1.89–1.75 (4 H, m), 1.56–1.48 (2 H, m), 1.31–1.22 (2 H, m), 0.84 (3 H, t, *J* = 7.37); ^13^C-NMR: 199.18, 148.77, 134.60, 128.67, 128.18, 37.31, 35.69, 33.38, 33.26, 32.29, 22.89, 22.35, 13.93.

*5-Bromo-1-(4-iso-butylphenyl)pentan-1-one* (**1o**). Yield: 51 %; ^1^H-NMR: 7.87 (2 H, d, *J* = 8.21), 7.23 (2 H, d, *J* = 8.14), 3.46–3.43 (2 H, m), 3.00–2.97 (2 H, m), 2.53 (2 H, d, *J* = 7.22), 1.97–1.86 (5 H, m), 0.90 (6 H, d, *J* = 6.65); ^13^C-NMR: 199.29, 147.61, 134.66, 129.37, 128.03, 45.41, 37.34, 33.42, 32.30, 30.15, 22.89, 22.38.

*5-Bromo-1-(4-tert-butylphenyl)pentan-1-one* (**1p**). Yield: 58 %; ^1^H-NMR: 7.81 (2 H, d, *J* = 8.66), 7.39 (2 H, d, *J* = 8.67), 3.37–3.34 (2 H, m), 2.91–2.88 (2 H, m), 1.90–1.77 (4 H, m), 1.25 (9 H, s); ^13^C-NMR: 199.22, 156.81, 134.31, 128.02, 125.58, 37.32, 35.12, 33.38, 32.27, 31.11, 22.89.

*4-Bromo-1-phenylbutan-1-one* (**1q**). Yield: 38 %; ^1^H-NMR: 7.85–7.83 (2 H, m), 7.45–7.41 (1 H, m), 7.34–7.31 (2 H, m), 3.41 (2 H, t, *J* = 6.38), 3.03 (2 H, t, *J* = 6.93), 2.19–2.14 (2 H, m) [Lit. [12] CDCl_3_: 8.00–7.96 (2 H, m), 7.60–7.50 (1 H, m), 7.48–7.44 (2 H, m), 3.55 (2 H, t, *J* = 6.60), 3.19 (2 H, t, *J* = 6.60), 2.36–2.27 (2 H, m)]; ^13^C-NMR: 198.36, 136.43, 132.93, 128.37, 127.72, 36.30, 33.43, 26.64.

*4-Bromo-1-(4-methylphenyl)butan-1-one* (**1r**). Yield: 52 %; ^1^H-NMR: 7.85 (2 H, d, *J* = 8.21), 7.23 (2 H, d, *J* = 7.92), 3.51 (2 H, t, *J* = 6.43), 3.11 (2 H, t, *J* = 6.98), 2.38 (3 H, s), 2.29–2.24 (2 H, m); ^13^C-NMR: 198.14, 143.80, 134.09, 129.13, 127.93, 36.23, 33.54, 26.82, 21.48.

*4-Bromo-1-(4-ethylphenyl)butan-1-one* (**1s**). Yield: 56 %; ^1^H-NMR: 7.90 (2 H, d, *J* = 8.24), 7.28 (2 H, d, *J* = 8.21), 3.54 (2 H, t, *J* = 6.44), 3.15 (2 H, t, *J* = 6.98), 2.73–2.68 (2 H, m), 2.32–2.27 (2 H, m), 1.26 (3 H, t, *J* = 7.68); ^13^C-NMR: 198.32, 150.15, 134.51, 128.25, 128.14, 36.47, 33.74, 28.97, 27.04, 15.25.

*4-Bromo-1-(4-n-propylphenyl)butan-1-one* (**1t**). Yield: 56 %; ^1^H-NMR: 7.91 (2 H, d, *J* = 8.26), 7.28 (2 H, d, *J* = 8.31), 3.55 (2 H, t, *J* = 6.39), 3.16 (2 H, t, *J* = 6.95), 2.67–2.64 (2 H, m), 2.33–2.28 (2 H, m), 1.71–1.64 (2 H, m), 0.96 (3 H, t, *J* = 7.40); ^13^C-NMR: 198.22, 148.50, 134.37, 128.58, 127.99, 37.87, 36.30, 33.57, 26.86, 24.08, 13.63.

*4-Bromo-1-(4-iso-propylphenyl)butan-1-one* (**1u**). Yield: 70 %; ^1^H-NMR: 7.93 (2 H, d, *J* = 8.34), 7.33 (2 H, d, *J* = 8.26), 3.55 (2 H, t, *J* = 6.38), 3.17 (2 H, t, *J* = 6.93), 3.01–2.95 (1 H, m), 2.34–2.29 (2 H, m), 1.28 (6 H, d, *J* = 6.93); ^13^C-NMR: 198.39, 154.74, 134.68, 128.29, 126.73, 36.46, 34.27, 33.68, 27.03, 23.68.

*4-Bromo-1-(4-n-butylphenyl)butan-1-one* (**1v**). Yield: 38 %; ^1^H-NMR: 7.80 (2 H, d, *J* = 8.30), 7.17 (2 H, d, *J* = 8.36), 3.45 (2 H, t, *J* = 6.40), 3.05 (2 H, t, *J* = 6.94), 2.58–2.55 (2 H, m), 2.22–2.17 (2 H, m), 1.55–1.49 (2 H, m), 1.30–1.22 (2 H, m), 0.84 (3 H, t, *J* = 7.38); ^13^C-NMR: 198.34, 148.90, 134.52, 128.68, 128.16, 36.46, 35.69, 33.67, 33.23, 27.04, 22.33, 13.92.

*4-Bromo-1-(4-iso-butylphenyl)butan-1-one* (**1w**). Yield: 51 %; ^1^H-NMR: 7.88 (2 H, d, *J* = 8.25), 7.22 (2 H, d, *J* = 8.21), 3.53 (2 H, t, *J* = 6.39), 3.14 (2 H, t, *J* = 6.94), 2.52 (2 H, d, *J* = 7.20), 2.31–2.26 (2 H, m), 1.93–1.85 (1 H, m), 0.90 (6 H, d, *J* = 6.63); ^13^C-NMR: 198.32, 147.60, 134.45, 129.25, 127.90, 45.28, 36.35, 33.56, 29.99, 26.92, 22.23.

*4-Bromo-1-(4-tert-butylphenyl)butan-1-one* (**1x**). Yield: 47 %; ^1^H-NMR: 7.92 (2 H, d, *J* = 8.66), 7.49 (2 H, d, *J* = 8.66), 3.56–3.54 (2 H, m), 3.16 (2 H, t, *J* = 6.93), 2.34–2.28 (2 H, m), 1.35 (9 H, s); ^13^C-NMR: 198.46, 156.98, 134.21, 127.99, 125.58, 36.44, 35.12, 33.66, 31.08, 27.00.

*6-(4'-(Hydroxydiphenylmethyl)piperidin-1-yl)-1-phenylhexan-1-one* (**2a**). Yield: 22 %; mp 130 °C; IR 2942, 2363, 1680, 1596, 1448, 1318, 1180; ^1^H-NMR: 8.45 (1 H, s), 7.96–7.94 (2 H, m), 7.59–7.45 (7 H, m), 7.31–7.27 (4 H, m), 7.20–7.17 (2 H, m), 3.46 (2 H, d, *J* = 10.83), 3.00–2.97 (2 H, m), 2.85–2.83 (2 H, m), 2.62–2.59 (3 H, m), 2.17–2.14 (2 H, m), 1.79–1.76 (4 H, m), 1.61 (2 H, d, *J* = 13.78), 1.44–1.41 (2 H, m); ^13^C-NMR: 199.92, 167.85, 145.55, 136.88, 133.09, 128.63, 128.32, 128.01, 126.69, 125.58, 78.85, 52.58, 42.61, 37.98, 26.57, 23.93, 23.43; LC/MS-MS 442.11 (M+H^+^).

*6-(4'-(Hydroxydiphenylmethyl)piperidin-1-yl)-1-(4-methylphenyl)hexan-1-one* (**2b**). Yield: 26 %; mp: 169 °C; IR: 2934, 2364, 1684, 1604, 1447, 1383, 1344, 1320, 1179; ^1^H-NMR: 8.44 (1 H, s), 7.81 (2 H, d, *J* = 8.14), 7.47 (4 H, d, *J* = 7.70), 7.28–7.21 (6 H, m), 7.16–7.13 (2 H, m), 3.38 (2 H, d, *J* = 11.19), 2.92 (2 H, t, *J* = 7.09), 2.78–2.75 (2 H, m), 2.60–2.49 (3 H, m), 2.38 (3 H, s), 2.14–2.06 (2 H, m), 1.75–1.69 (4 H, m), 1.56 (2 H, d, *J* = 13.83), 1.41–1.34 (2 H, m); ^13^C-NMR: 199.64, 168.13, 145.62, 143.90, 134.42, 129.32, 128.32, 128.17, 126.69, 125.62, 78.90, 52.72, 42.70, 37.92, 26.68, 24.11, 23.60, 21.67; LC/MS-MS: 456.13 (M+H^+^).

*6-(4'-(Hydroxydiphenylmethyl)piperidin-1-yl)-1-(4-ethylphenyl)hexan-1-one* (**2c**). Yield: 36 %; mp: 174 °C; IR: 2939, 1685, 1605, 1447, 1383, 1345, 1320, 1286, 1148; ^1^H-NMR: 8.48 (1 H, s), 7.86 (2 H, d, *J* = 8.33), 7.51–7.49 (4 H, m), 7.30–7.26 (6 H, m), 7.19–7.16 (2 H, m), 3.38 (2 H, d, *J* = 11.17), 2.94 (2 H, t, *J* = 7.10), 2.77–2.68 (4 H, m), 2.63–2.45 (3 H, m), 2.15–2.05 (2 H, m), 1.78–1.69 (4 H, m), 1.58 (2 H, d, *J* = 13.44), 1.43–1.36 (2 H, m), 1.25 (3 H, t, *J* = 7.60); ^13^C-NMR: 199.65, 150.05, 145.62, 134.62, 128.31, 128.26, 128.12, 126.67, 125.59, 78.92, 42.73, 37.94, 28.95, 26.72, 24.23, 23.62, 15.23; LC/MS-MS: 470.18 (M+H^+^).

*6-(4'-(Hydroxydiphenylmethyl)piperidin-1-yl)-1-(4-n-propylphenyl)hexan-1-one* (**2d**). Yield: 43 %; mp: 172 °C; IR: 2940, 2395, 1686, 1605, 1447, 1384, 1345, 1318, 1286, 1177; ^1^H-NMR: 8.27 (1 H, s), 7.64 (2 H, d, *J* = 8.33), 7.30–7.28 (4 H, m), 7.09–7.03 (6 H, m), 6.96 (2 H, t, *J* = 7.34), 3.17 (2 H, d, *J* = 11.10), 2.73 (2 H, t, *J* = 7.11), 2.56–2.53 (2 H, m), 2.44–2.23 (5 H, m), 1.95–1.85 (2 H, m), 1.57–1.35 (8 H, m), 1.22–1.16 (2 H, m), 0.73 (3 H, t, *J* = 7.37); ^13^C-NMR: 199.64, 167.99, 148.52, 145.58, 128.69, 128.28, 128.12, 126.65, 125.55, 119.97, 52.75, 43.18, 42.71, 38.00, 37.91, 26.70, 24.21, 23.58, 13.75; LC/MS-MS: 484.24 (M+H^+^).

*6-(4'-(Hydroxydiphenylmethyl)piperidin-1-yl)-1-(4-iso-propylphenyl)hexan-1-one* (**2e**). Yield: 43 %; mp: 154 °C; IR: 2936, 2360, 1686, 1606, 1446, 1382, 1345, 1317, 1286, 1181; ^1^H-NMR (d_4_-MeOH): 8.53 (1 H, s), 7.91 (2 H, d, *J* = 8.35), 7.53–7.51 (4 H, m), 7.36 (2 H, d, *J* = 8.25), 7.31–7.28 (4 H, m), 7.19–7.16 (2 H, m), 3.51 (2 H, d, *J* = 12.19), 3.05–2.92 (7 H, m), 2.86–2.80 (1 H, m), 1.83–1.69 (8 H, m), 1.46–1.40 (2 H, m), 1.26 (6 H, d, *J* = 6.93); ^13^C-NMR (d_4_-MeOH): 202.03, 156.18, 147.20, 136.14, 129.54, 129.47, 129.25, 129.19, 127.87, 127.80, 127.67, 127.13, 79.87, 53.91, 42.96, 38.90, 35.51, 27.33, 25.50, 25.16, 24.80, 24.09; LC/MS-MS: 484.24 (M+H^+^).

*6-(4'-(Hydroxydiphenylmethyl)piperidin-1-yl)-1-(4-n-butylphenyl)hexan-1-one* (**2f**). Yield: 41 %; mp: 142 °C; IR: 2947, 2337, 1684, 1606, 1446, 1383, 1345, 1316, 1285, 1177; ^1^H-NMR (d_4_-MeOH): 8.53 (1 H, s), 7.90 (2 H, d, *J* = 8.27), 7.53–7.51 (4 H, m), 7.32–7.28 (6 H, m), 7.18 (2 H, t, *J* = 7.32), 3.52 (2 H, d, *J* = 12.35), 3.05–2.93 (6 H, m), 2.85–2.81 (1 H, m), 2.69–2.66 (2 H, m), 1.84–1.70 (8 H, m), 1.65–1.59 (2 H, m), 1.46–1.33 (4 H, m), 0.94 (3 H, t, *J* = 7.34); ^13^C-NMR (d_4_-MeOH): 204.60, 152.84, 149.71, 138.49, 132.38, 132.32, 131.92, 131.87, 131.70, 130.18, 129.63, 82.37, 56.42, 45.46, 39.13, 37.09, 29.81, 28.02, 27.66, 27.28, 25.88, 16.78; LC/MS-MS: 498.24 (M+H^+^).

*6-(4'-(Hydroxydiphenylmethyl)piperidin-1-yl)-1-(4-iso-butylphenyl)hexan-1-one* (**2g**). Yield: 43 %; mp: 201 °C; IR: 2940, 2357, 1684, 1605, 1446, 1385, 1345, 1286, 1177; ^1^H-NMR (d_4_-MeOH): 8.54 (1 H, s), 7.91 (2 H, d, *J* = 8.29), 7.54–7.52 (4 H, m), 7.32–7.29 (6 H, m), 7.20–7.17 (2 H, m), 3.52 (2 H, d, *J* = 12.22), 3.06–2.94 (6 H, m), 2.86–2.81 (1 H, m), 2.56 (2 H, d, *J* = 7.22), 1.96–1.72 (10 H, m), 1.48–1.42 (2 H, m), 0.92 (6 H, d, *J* = 6.63); ^13^C-NMR (d_4_-MeOH): 200.20, 149.13, 147.16, 136.06, 130.54, 129.18, 127.68, 127.12, 79.88, 53.96, 46.29, 42.97, 31.35, 27.27, 25.55, 25.52, 25.18, 24.71, 22.67; LC/MS-MS: 498.23 (M+H^+^).

*6-(4'-(Hydroxydiphenylmethyl)piperidin-1-yl)-1-(4-tert-butylphenyl)hexan-1-one* (**2h**). Yield 56 %; mp 67 °C; IR 2962, 2359, 1677, 1603, 1447, 1271, 1191; ^1^H-NMR (d_4_-MeOH) 8.48 (1 H, s), 7.92 (2 H, d, *J* = 8.49), 7.54–7.51 (6 H, m), 7.31–7.28 (4 H, m), 7.18 (2 H, t, *J* = 7.30), 3.53 (2 H, d, *J* = 12.10), 3.07–2.96 (6 H, m), 2.86–2.81 (1 H, m), 1.84–1.74 (8 H, m), 1.47–1.41 (2 H, m), 1.34 (9 H, s); ^13^C-NMR (d_4_-MeOH) 202.08, 158.30, 147.16, 135.67, 129.19, 127.65, 127.11, 126.73, 112.07, 79.86, 53.87, 42.89, 38.89, 36.00, 31.49, 27.26, 25.07, 24.80, 24.77; LC/MS-MS 498.23 (M+H^+^).

*5-(4’-(Hydroxydiphenylmethyl)piperidin-1-yl)-1-phenylpentan-1-one* (**2i**). Yield: 25 %; mp: 143 °C; IR: 3059, 2954, 2361, 1682, 1595, 1446, 1388, 1341, 1206; ^1^H-NMR: 8.31 (1 H, s), 7.81–7.79 (2 H, m), 7.45–7.42 (1 H, m), 7.38–7.37 (4 H, m), 7.35–7.32 (2 H, m), 7.17–7.14 (4 H, m), 7.06–7.03 (2 H, m), 3.33 (2 H, d, *J* = 11.67), 2.92–2.90 (2 H, m), 2.76–2.73 (2 H, m), 2.51–2.43 (3 H, m), 2.05–1.96 (2 H, m), 1.70–1.61 (4 H, m), 1.47 (2 H, d, *J* = 14.19); ^13^C-NMR: 199.46, 145.57, 136.68, 133.22, 128.66, 128.30, 127.98, 126.67, 125.58, 78.84, 52.64, 42.63, 37.60, 23.76, 21.24; LC/MS-MS: 428.10 (M+H^+^).

*5-(4’-(Hydroxydiphenylmethyl)piperidin-1-yl)-1-(4-methylphenyl)pentan-1-one* (**2j**). Yield: 40 %; mp: 165 °C; IR: 2953, 2358, 1679, 1592, 1445, 1387, 1343, 1318, 1180; ^1^H-NMR (CDCl_3_ + d_4_-MeOH): 8.44 (1 H, s), 7.76 (2 H, d, *J* = 8.28), 7.43 (4 H, d, *J* = 7.28), 7.23–7.17 (6 H, m), 7.11–7.07 (2 H, m), 3.42 (2 H, d, *J* = 12.05), 2.97–2.85 (4 H, m), 2.58–2.52 (3 H, m), 2.33 (3 H, s), 2.03–1.90 (2 H, m), 1.72–1.67 (4 H, m), 1.52 (2 H, d, *J* = 14.56); ^13^C-NMR (CDCl_3_ + d_4_-MeOH): 199.21, 168.45, 145.32, 143.90, 133.78, 129.02, 127.93, 127.79, 126.25, 125.16, 78.01, 52.32, 42.18, 37.02, 23.13, 23.11, 23.06, 21.26, 20.79, 20.74; LC/MS-MS: 442.20 (M+H^+^).

*5-(4’-(Hydroxydiphenylmethyl)piperidin-1-yl)-1-(4-ethylphenyl)pentan-1-one* (**2k**). Yield: 30 %; mp: 167 °C; IR 2953, 2362, 1678, 1605, 1446, 1388, 1342, 1318, 1178; ^1^H-NMR (CDCl_3_ + d_4_-MeOH): 8.38 (1 H, s), 7.78 (2 H, d, *J* = 8.34), 7.42 (4 H, d, *J* = 9.52), 7.23–7.19 (6 H, m), 7.11–7.08 (2 H, m), 3.36 (2 H, d, *J* = 11.60), 2.94–2.91 (2 H, m), 2.79–2.75 (2 H, m), 2.63 (2 H, q, *J* = 7.60), 2.56–2.40 (3 H, m), 2.03 (2 H, d, *J* = 12.51), 1.72–1.69 (4 H, m), 1.52 (2 H, d, *J* = 13.92), 1.18 (3 H, t, *J* = 7.62); ^13^C-NMR (CDCl_3_ + d_4_-MeOH): 199.16, 168.05, 150.19, 145.59, 134.44, 128.29, 128.21, 128.14, 126.65, 125.58, 78.86, 52.66, 42.67, 37.52, 30.92, 28.92, 23.87, 23.53, 21.38, 15.17; LC/MS-MS: 465.20 (M+H^+^).

*5-(4’-(Hydroxydiphenylmethyl)piperidin-1-yl)-1-(4-n-propylphenyl)pentan-1-one* (**2l**). Yield: 37 %; mp: 166 °C; IR: 3059, 2954, 2357, 1679, 1605, 1446, 1384, 1341, 1315, 1179 ^1^H-NMR: 8.37 (1 H, s), 7.77 (2 H, d, *J* = 8.24), 7.42 (4 H, d, *J* = 7.91), 7.22–7.16 (6 H, m), 7.10–7.07 (2 H, m), 3.34 (2 H, d, *J* = 11.46), 2.93–2.90 (2 H, m), 2.75–2.73 (2 H, m), 2.57–2.42 (5 H, m), 2.06–1.97 (2 H, m), 1.68–1.48 (8 H, m), 0.86 (3 H, t, *J* = 7.31); ^13^C-NMR: 199.19, 168.15, 148.67, 145.67, 134.46, 128.74, 128.26, 128.12, 126.62, 125.61, 78.84, 52.62, 42.70, 38.00, 37.54, 24.19, 23.89, 23.57, 21.40, 13.75; LC/MS-MS: 470.22 (M+H^+^).

*5-(4’-(Hydroxydiphenylmethyl)piperidin-1-yl)-1-(4-iso-propylphenyl)pentan-1-one* (**2m**). Yield: 33 %; mp: 166 °C; IR: 2963, 2360, 1679, 1596, 1447, 1389, 1342, 1317, 1188; ^1^H-NMR: 8.37 (1 H, s), 7.80 (2 H, d, *J* = 8.20), 7.42 (4 H, d, *J* = 8.20), 7.23–7.19 (6 H, m), 7.11–7.08 (2 H, m), 3.34 (2 H, d, *J* = 11.98), 2.93–2.85 (3 H, m), 2.78–2.75 (2 H, m), 2.54–2.44 (3 H, m), 2.06–1.99 (2 H, m), 1.69–1.68 (4 H, m), 1.51 (2 H, d, *J* = 13.87), 1.18 (6 H, d, *J* = 6.94); ^13^C-NMR: 199.17, 168.13, 154.75, 145.63, 134.58, 128.29, 128.26, 126.73, 125.59, 126.64, 78.85, 42.68, 37.53, 34.24, 23.86, 23.65, 21.38; LC/MS-MS: 470.30 (M+H^+^).

*5-(4’-(Hydroxydiphenylmethyl)-piperidin-1-yl)-1-(4-n-butylphenyl)pentan-1-one* (**2n**). Yield: 28 %; mp: 157 °C; IR: 2955, 2365, 1679, 1604, 1447, 1387, 1341, 1314, 1248, 1183; ^1^H-NMR: 8.37 (1 H, s), 7.77 (2 H, d, *J* = 8.24), 7.42 (4 H, d, *J* = 7.41), 7.22–7.17 (6 H, m), 7.11–7.07 (2 H, m), 3.37 (2 H, d, *J* = 11.56), 2.94–2.91 (2 H, m), 2.79–2.76 (2 H, m), 2.60–2.45 (5 H, m), 2.09–2.00 (2 H, m), 1.71–1.65 (4 H, m), 1.57–1.49 (4 H, m), 1.32–1.24 (2 H, m), 0.85 (3 H, t, *J* = 7.29); ^13^C-NMR: 199.17, 168.09, 148.96, 145.62, 134.41, 128.70, 128.30, 128.14, 126.65, 125.59, 78.85, 52.64, 42.66, 37.52, 35.69, 33.23, 23.84, 23.51, 22.32, 21.37, 13.91; LC/MS-MS: 484.34 (M+H^+^).

*5-(4’-(Hydroxydiphenylmethyl)-piperidin-1-yl)-1-(4-iso-butylphenyl)pentan-1-one* (**2o**). Yield: 29 %; mp: 173 °C; IR: 2953, 2365, 1679, 1605, 1447, 1387, 1341, 1318, 1234, 1187; ^1^H-NMR: 8.47 (1 H, s), 7.87–7.85 (2 H, m), 7.52–7.50 (4 H, m), 7.30–7.19 (8 H, m), 3.45–3.43 (2 H, m), 3.03–3.01 (2 H, m), 2.85–2.84 (2 H, m), 2.64–2.53 (5 H, m), 2.16–2.11 (2 H, m), 1.94–1.89 (1 H, m), 1.78 (4 H, m), 1.60 (2 H, d, *J* = 13.64), 0.93–0.91 (6 H, m); ^13^C-NMR: 199.19, 167.96, 147.75, 145.56, 134.51, 129.38, 128.32, 127.99, 126.69, 125.58, 78.90, 52.69, 45.39, 42.71, 37.53, 30.10, 23.91, 23.57, 22.34, 21.41; LC/MS-MS: 484.34 (M+H^+^).

*5-(4’-(Hydroxydiphenylmethyl)-piperidin-1-yl)-1-(4-tert-butylphenyl)pentan-1-one* (**2p**). Yield: 26 %; mp: 171 °C; IR: 2964, 2365, 1684, 1588, 1446, 1380, 1342, 1322, 1230, 1188; ^1^H-NMR: 8.38 (1 H, s), 7.81–7.79 (2 H, m), 7.43–7.39 (6 H, m), 7.23–7.20 (4 H, m), 7.12–7.09 (2 H, m), 3.37–3.35 (2 H, d, *J* = 10.49), 2.94–2.92 (2 H, m), 2.77–2.76 (2 H, m), 2.55–2.45 (3 H, m), 2.08–2.01 (1 H, m), 1.70 (4 H, m), 1.52 (2 H, d, *J* = 14.12), 1.26 (9 H, s); ^13^C-NMR: 199.16, 167.91, 156.99, 145.55, 134.16, 128.32, 127.97, 126.70, 125.60, 125.58, 78.90, 52.73, 52.69, 42.70, 37.54, 35.13, 31.09, 23.89, 23.58, 23.55, 21.42; LC/MS-MS: 484.31 (M+H^+^).

*4-(4’-(Hydroxydiphenylmethyl)piperidin-1-yl)-1-phenylbutan-1-one* (**2q**). Yield: 18 %; mp: 175 °C; IR: 2358, 1681, 1596, 1489, 1447, 1365, 1345, 1065; ^1^H-NMR (d_4_-MeOH): 8.53 (1 H, s), 8.00–7.99 (2 H, m), 7.61 (1 H, t, *J* = 7.41), 7.54–7.49 (6 H, m), 7.32–7.28 (4 H, m), 7.18 (2 H, t, *J* = 7.36), 3.57 (2 H, d, *J* = 12.26), 3.20–3.17 (2 H, m), 3.12–3.09 (2 H, m), 3.01–2.96 (2 H, m), 2.88–2.82 (1 H, m), 2.13–2.07 (2 H, m), 1.86–1.72 (4 H, m) [Lit. [14] 200 MHz, CDCl_3_: 7.90–7.05 (15 H, m), 2.89 (4 H, m), 2.31 (3 H, m), 1.98–1.77 (4 H, m), 1.52–1.20 (4 H, m)]; ^13^C-NMR (d_4_-MeOH): 198.98, 145.82, 136.52, 133.16, 128.42, 127.79, 127.71, 126.26, 125.71, 78.49, 55.98, 52.63, 47.64, 41.59, 34.77, 24.17, 18.35; LC/MS-MS: 414.18 (M+H^+^).

*4-(4’-(Hydroxydiphenylmethyl)piperidin-1-yl)-1-(4-methylphenyl)butan-1-one* (**2r**). Yield: 13 %; mp: 191 °C; IR: 2358, 1681, 1596, 1489, 1447, 1365, 1345, 1065; ^1^H-NMR (d_4_-MeOH): 8.53 (1 H, s), 7.89 (2 H, d, *J* = 8.24), 7.54–7.52 (4 H, m), 7.32–7.28 (6 H, m), 7.18 (2 H, t, *J* = 7.35), 3.56 (2 H, d, *J* = 12.32), 3.16–3.08 (4 H, m), 3.00–2.95 (2 H, m), 2.88–2.82 (1 H, m), 2.40 (3 H, s), 2.10–2.06 (2 H, m), 1.86–1.71 (4 H, m); ^13^C-NMR (d_4_-MeOH): 200.22, 147.24, 145.74, 133.45, 130.44, 129.30, 129.21, 127.67, 127.12, 79.90, 54.03, 43.00, 25.57, 21.66, 19.76, 19.70; LC/MS-MS: 428.32 (M+H^+^) [Lit. [15] 428 (M+H^+^)].

*4-(4’-(Hydroxydiphenylmethyl)piperidin-1-yl)-1-(4-ethylphenyl)butan-1-one* (**2s**). Yield: 20 %; mp: 204 °C; IR: 2968, 2358, 1687, 1606, 1446, 1388, 1346, 1183; ^1^H-NMR (d_4_-MeOH): 8.52 (1 H, s), 7.92 (2 H, d, *J* = 8.33), 7.54–7.52 (4 H, m), 7.35–7.28 (6 H, m), 7.19–7.16 (2 H, m), 3.56 (2 H, d, *J* = 12.34), 3.17–3.08 (4 H, m), 3.01–2.95 (2 H, m), 2.87–2.81 (1 H, m), 2.74–2.69 (2 H, m), 2.12–2.06 (2 H, m), 1.85–1.72 (4 H, m), 1.25 (3 H, t, *J* = 7.62); ^13^C-NMR (d_4_-MeOH): 200.19, 151.95, 147.19, 135.67, 129.40, 129.28, 129.18, 127.67, 127.12, 79.91, 54.09, 43.02, 36.08, 29.89, 25.62, 19.87, 15.70; LC/MS-MS: 442.32 (M+H^+^) [Lit. [15] 442 (M+H^+^)].

*4-(4’-(Hydroxydiphenylmethyl)piperidin-1-yl)-1-(4-n-propylphenyl)butan-1-one* (**2t**). Yield: 10 %; mp: 182 °C; IR: 3182, 2359, 1684, 1602, 1446, 1388, 1347; ^1^H-NMR (d_4_-MeOH): 8.54 (1 H, s), 7.92 (2 H, d, *J* = 8.31), 7.55–7.53 (4 H, m), 7.34–7.29 (6 H, m), 7.20–7.17 (2 H, t, *J* = 7.35), 3.56 (2 H, d, *J* = 12.20), 3.18–3.08 (4 H, m), 3.00–2.95 (2 H, m), 2.87–2.82 (1 H, m), 2.69–2.66 (2 H, m), 2.11–2.07 (2 H, m), 1.83–1.64 (6 H, m), 0.95 (3 H, t, *J* = 7.38); ^13^C-NMR (d_4_-MeOH): 200.21, 150.33, 147.21, 135.71, 129.91, 129.31, 129.18, 127.66, 127.12, 79.91, 57.50, 54,08, 43.04, 38.96, 36.10, 25.62, 25.37, 19.89, 19.83, 14.02; LC/MS-MS: 456.24 (M+H^+^) [Lit. [15] 456 (M+H^+^)].

*4-(4’-(Hydroxydiphenylmethyl)piperidin-1-yl)-1-(4-iso-propylphenyl)butan-1-one* (**2u**). Yield: 32 %; mp: 189 °C; IR: 2960, 2366, 1691, 1596, 1446, 1345, 1189, 1062; ^1^H-NMR (d_4_-MeOH): 8.52 (1 H, s), 7.92 (2 H, d, *J* = 8.38), 7.54–7.52 (4 H, m), 7.37 (2 H, d, *J* = 8.26), 7.30 (4 H, t, *J* = 7.76), 7.19–7.16 (2 H, m), 3.56 (2 H, d, *J* = 12.30), 3.17–3.08 (4 H, m), 3.00–2.95 (3 H, m), 2.87–2.81 (1 H, m), 2.11–2.06 (2 H, m), 1.85–1.72 (4 H, m), 1.27 (6 H, t, *J* = 6.92); ^13^C-NMR (d_4_-MeOH): 200.24, 156.44, 147.21, 135.82, 129.45, 129.19, 127.86, 127.67, 127.12, 79.90, 57.43, 54,06, 43.02, 36.10, 35.53, 25.59, 24.04, 19.80; LC/MS-MS: 456.38 (M+H^+^).

*4-(4’-(Hydroxydiphenylmethyl)piperidin-1-yl)-1-(4-n-butylphenyl)butan-1-one* (**2v**). Yield: 6 %; mp: 161 °C; IR: 2963, 1687, 1607, 1448, 1362, 1182, 1139; ^1^H-NMR (d_4_-MeOH): 8.54 (1 H, s), 7.91 (2 H, d, *J* = 8.30), 7.54–7.52 (4 H, m), 7.33–7.28 (6 H, m), 7.18 (2 H, t, *J* = 7.33), 7.19–7.16 (2 H, m), 3.55 (2 H, d, *J* = 12.29), 3.16–3.06 (4 H, m), 2.98–2.93 (2 H, m), 2.87–2.81 (1 H, m), 2.70–2.67 (2 H, m), 2.12–2.06 (2 H, m), 1.85–1.71 (4 H, m), 1.65–1.59 (2 H, m), 1.40–1.32 (2 H, m), 0.94 (3 H, t, *J* = 7.40); ^13^C-NMR (d_4_-MeOH): 200.23, 150.56, 147.23, 135.67, 129.85, 129.32, 129.18, 127.65, 127.12, 79.92, 57.50, 54,08, 43.07, 36.61, 36.11, 34.52, 25.62, 23.33, 19.91, 14.22; LC/MS-MS: 470.17 (M+H^+^).

*4-(4’-(Hydroxydiphenylmethyl)piperidin-1-yl)-1-(4-iso-butylphenyl)butan-1-one* (**2w**). Yield: 20 %; mp: 155 °C; IR: 2956, 1689, 1605, 1448, 1385, 1316, 1193; ^1^H-NMR (d_4_-MeOH): 8.55 (1 H, s), 7.93 (2 H, d, *J* = 8.28), 7.55–7.53 (4 H, m), 7.33–7.29 (6 H, m), 7.19 (2 H, t, *J* = 7.33), 3.56 (2 H, d, *J* = 12.35), 3.18–3.08 (4 H, m), 3.00–2.95 (2 H, m), 2.88–2.83 (1 H, m), 2.56 (2 H, d, *J* = 7.23), 2.13–2.07 (2 H, m), 1.96–1.73 (5 H, m), 0.92 (6 H, d, *J* = 6.63); ^13^C-NMR (d_4_-MeOH): 195.87, 149.37, 147.20, 135.75, 130.54, 129.18, 127.66, 127.12, 79.92, 54.11, 46.30, 43.05, 36.10, 31.35, 25.66, 22.66, 19.90, 19.85; LC/MS-MS: 470.17 (M+H^+^).

*4-(4’-(Hydroxydiphenylmethyl)piperidin-1-yl)-1-(4-tert-butylphenyl)butan-1-one* (**2x**). Yield: 27 %; mp: 175 °C; IR: 2961, 2330, 1691, 1590, 1447, 1383, 1346, 1192; ^1^H-NMR (d_4_-MeOH): 8.39 (1 H, s), 7.93 (2 H, d, *J* = 8.58), 7.55–7.53 (6 H, m), 7.31–7.28 (4 H, m), 7.19–7.16 (2 H, m), 3.59 (2 H, d, *J* = 12.22), 3.18–3.12 (4 H, m), 3.04–3.00 (2 H, m), 2.89–2.84 (1 H, m), 2.13–2.07 (2 H, m), 1.89–1.80 (2 H, m), 1.74–1.71 (2 H, m), 1.34 (9 H, s); ^13^C-NMR (d_4_-MeOH): 200.12, 168.28, 158.52, 147.21, 135.34, 129.21, 127.67, 127.10, 126.75, 79.87, 53.88, 42.88, 36.08, 36.02, 31.49, 25.40, 19.66; LC/MS-MS: 470.03 (M+H^+^) [Lit. 470 (M+H^+^)] [15].

*6-(4'-(Hydroxydiphenylmethyl)piperidin-1-yl)-1-phenylhexan-1-ol* (**3a**). Yield: 83 %; mp: 80 °C; IR: 2941, 2503, 1492, 1448, 1345, 1066; ^1^H-NMR (d_4_-MeOH): 7.52 (4 H, d, *J* = 7.80), 7.32–7.28 (8 H, m), 7.24–7.21 (1 H, m), 7.19–7.16 (2 H, m), 4.61–4.59 (1 H, m), 3.52 (2 H, d, *J* = 12.23), 3.03–2.96 (4 H, m), 2.88–2.82 (1 H, m), 1.82–1.66 (8 H, m), 1.49–1.29 (4 H, m); ^13^C-NMR (d_4_-MeOH): 146.97, 146.31, 129.16, 129.05, 128.12, 127.53, 126.95, 126.91, 79.65, 74.79, 58.06, 53.90, 42.67, 39.71, 27.44, 26.21, 25.61, 24.93; LC/MS-MS: 444.21 (M+H^+^).

*6-(4'-(Hydroxydiphenylmethyl)piperidin-1-yl)-1-(4-methylphenyl)hexan-1-ol* (**3b**). Yield: 62 %; mp: 71 °C; IR: 2941, 2361, 1593, 1491, 1447, 1335, 1066; ^1^H-NMR (d_4_-MeOH): 8.53 (1 H, s), 7.51 (4 H, d, *J* = 7.39), 7.31–7.28 (4 H, m), 7.21–7.16 (4 H, m), 7.12 (2 H, d, *J* = 7.92), 4.55 (1 H, t, *J* = 6.62), 3.48 (2 H, d, *J* = 12.38), 2.98–2.89 (4 H, m), 2.84–2.78 (1 H, m), 2.30 (3 H, s), 1.81–1.63 (8 H, m), 1.47–1.27 (4 H, m); ^13^C-NMR (d_4_-MeOH): 170.17, 147.20, 143.39, 137.98, 129.91, 129.20, 127.68, 127.12, 127.07, 79.88, 74.85, 53.92, 42.99, 39.82, 27.68, 26.42, 25.56, 25.53, 25.24, 21.17; LC/MS-MS: 458.21 (M+H^+^).

*6-(4'-(Hydroxydiphenylmethyl)piperidin-1-yl)-1-(4-ethylphenyl)hexan-1-ol* (**3c**). Yield: 82 %; mp: 75 °C; IR: 2936, 2364, 1595, 1447, 1359, 1066; ^1^H-NMR (d_4_-MeOH): 8.53 (1 H, s), 7.53–7.51 (4 H, m), 7.31–7.27 (4 H, m), 7.22 (2 H, d, *J* = 8.04), 7.19–7.14 (4 H, m), 4.56 (1 H, t, *J* = 6.62), 3.48 (2 H, d, *J* = 12.67), 2.99–2.90 (4 H, m), 2.85–2.79 (1 H, m), 2.63–2.59 (2 H, q, *J* = 7.61), 1.83–1.63 (8 H, m), 1.47–1.27 (4 H, m), 1.21 (3 H, t, *J* = 7.59); ^13^C-NMR (d_4_-MeOH): 147.21, 144.55, 143.66, 129.20, 128.76, 127.67, 127.15, 127.11, 79.87, 74.87, 53.87, 42.95, 39.82, 29.57, 27.67, 26.43, 25.50, 25.18, 16.32; LC/MS-MS: 472.21 (M+H^+^).

*6-(4'-(Hydroxydiphenylmethyl)piperidin-1-yl)-1-(4-n-propylphenyl)hexan-1-ol* (**3d**). Yield: 71 %; mp: 73 °C; IR: 2933, 2364, 1596, 1448, 1334, 1179, 1064; ^1^H-NMR (d_4_-MeOH): 8.54 (1 H, s), 7.52–7.51 (4 H, m), 7.31–7.27 (4 H, m), 7.22 (2 H, d, *J* = 8.05), 7.19–7.12 (4 H, m), 4.56 (1 H, t, *J* = 6.55), 3.45 (2 H, d, *J* = 12.32), 2.95–2.78 (5 H, m), 2.57–2.54 (2 H, m), 1.80–1.58 (10 H, m), 1.46–1.28 (4 H, m), 0.92 (3 H, t, *J* = 7.38); ^13^C-NMR (d_4_-MeOH): 145.80, 142.28, 141.43, 127.98, 127.76, 126.23, 125.69, 125.63, 120.00, 79.47, 73.45, 56.54, 52.53, 41.65, 38.39, 37.32, 26.28, 25.03, 24.41, 24.17, 23.88, 12.68; LC/MS-MS: 486.27 (M+H^+^).

*6-(4'-(Hydroxydiphenylmethyl)piperidin-1-yl)-1-(4-iso-propylphenyl)hexan-1-ol* (**3e**). Yield: 75 %; mp: 62 °C; IR: 2961, 2364, 1597, 1448, 1333, 1180, 1064; ^1^H-NMR (d_4_-MeOH): 8.53 (1 H, s), 7.51 (4 H, d, *J* = 8.15), 7.31–7.28 (4 H, m), 7.23 (2 H, d, *J* = 8.12), 7.19–7.16 (4 H, m), 4.58–4.55 (1 H, m), 3.47 (2 H, d, *J* = 11.87), 2.98–2.79 (6 H, m), 1.80–1.63 (7 H, m), 1.47–1.24 (4 H, m), 1.23 (6 H, d, *J* = 6.93); ^13^C-NMR (d_4_-MeOH): 149.17, 147.19, 143.81, 129.20, 127.68, 127.29, 127.12, 79.88, 74.86, 53.91, 42.98, 39.81, 35.14, 27.68, 26.45, 25.54, 25.23, 24.54; LC/MS-MS: 486.34 (M+H^+^).

*6-(4'-(Hydroxydiphenylmethyl)piperidin-1-yl)-1-(4-n-butylphenyl)hexan-1-ol* (**3f**). Yield: 55 %; mp: 64 °C; IR 2930, 2356, 1595, 1448, 1341, 1068; ^1^H-NMR (d_4_-MeOH): 8.54 (1 H, s), 7.51 (4 H, d, *J* = 7.37), 7.31–7.27 (4 H, m), 7.22 (2 H, d, *J* = 8.04), 7.19–7.12 (4 H, m), 4.56 (1 H, m), 3.44 (2 H, d, *J* = 12.00), 2.93–2.76 (5 H, m), 2.58 (2 H, t, *J* = 7.72), 1.79–1.54 (10 H, m), 1.46–1.29 (6 H, m), 0.93 (3 H, t, *J* = 7.40); ^13^C-NMR (d_4_-MeOH): 147.24, 143.66, 143.08, 129.35, 129.19, 127.67, 127.14, 79.91, 74.90, 54.01, 43.14, 39.81, 36.33, 35.07, 27.74, 26.47, 25.67, 25.38, 23.36, 14.32; LC/MS-MS: 500.45 (M+H^+^).

*6-(4'-(Hydroxydiphenylmethyl)piperidin-1-yl)-1-(4-iso-butylphenyl)hexan-1-ol* (**3g**). Yield: 76 %; mp: 70 °C; IR: 2953, 1596, 1447, 1339, 1168, 1065; ^1^H-NMR (d_4_-MeOH): 8.55 (1 H, s), 7.53–7,52 (4 H, m), 7.32–7.29 (4 H, m), 7.25–7.17 (4 H, m), 7.11 (2 H, d, *J* = 8.05), 4.59–4.57 (1 H, m), 3.48 (2 H, d, *J* = 12.41), 2.98–2.79 (5 H, m), 2.47–2.45 (2 H, d, *J* = 7.18), 1.87–1.64 (9 H, m), 1.49–1.29 (4 H, m), 0.90 (6 H, d, *J* = 6.62); ^13^C-NMR (d_4_-MeOH): 147.17, 143.76, 141.87, 130.05, 129.18, 127.67, 127.11, 126.93, 79.89, 74.88, 53.94, 46.13, 43.01, 39.76, 31.50, 27.66, 26.43, 25.25, 22.71, 19.23; LC/MS-MS: 500.19 (M+H^+^).

*6-(4'-(Hydroxydiphenylmethyl)piperidin-1-yl)-1-(4-tert-butylphenyl)hexan-1-ol* (**3h**). Yield: 69 %; mp: 73 °C; IR: 2957, 2365, 1597, 1447, 1341, 1171, 1066; ^1^H-NMR (d_4_-MeOH): 8.52 (1 H, s), 7.53–7.52 (4 H, m), 7.37 (2 H, d, *J* = 8.41), 7.32–7.25 (6 H, m), 7.20–7.17 (2 H, m), 4.59–4.57 (1 H, m), 3.50 (2 H, d, *J* = 12.34), 3.02–2.94 (4 H, m), 2.86–2.80 (1 H, m), 1.83–1.65 (8 H, m), 1.49–1.32 (4 H, m), 1.31 (9 H, s); ^13^C-NMR (d_4_-MeOH): 169.85, 151.33, 147.13, 143.35, 129.20, 127.69, 127.10, 126.84, 126.16, 79.88, 74.76, 53.87, 49.04, 42.90, 39.74, 35.32, 31.85, 27.63, 26.41, 25.14; LC/MS-MS: 500.33 (M+H^+^).

*5-(4’-(Hydroxydiphenylmethyl)piperidin-1-yl)-1-phenylpentan-1-ol* (**3i**). Yield: 75 %; mp: 182 °C; IR: 2778, 2361, 1629, 1448, 1356, 1064; ^1^H-NMR (d_4_-MeOH): 8.55 (2 H, s), 7.52–7.50 (4 H, m), 7.34–7.27 (8 H, m), 7.24–7.20 (1 H, m), 7.18–7.15 (2 H, m), 4.61 (1 H, t, *J* = 5.78), 3.45 (2 H, d, *J* = 12.37), 2.95–2.76 (5 H, m), 1.84–1.65 (8 H, m), 1.51–1.29 (3 H, m); ^13^C-NMR (d_4_-MeOH): 147.20, 146.29, 129.34, 129.15, 128.32, 127.63, 127.11, 127.02, 79.89, 74.71, 53.93, 43.11, 39.53, 25.56, 25.28, 24.13; LC/MS-MS: 430.14 (M+H^+^).

*5-(4’-(Hydroxydiphenylmethyl)piperidin-1-yl)-1-(4-methylphenyl)pentan-1-ol* (**3j**). Yield: 71 %; mp: 84 °C; IR: 3340, 2951, 2361, 1595, 1491, 1447, 1330, 1173; ^1^H-NMR: 8.41 (1 H, s), 7.49 (4 H, d, *J* = 7.53), 7.29–7.25 (4 H, m), 7.20–7.09 (6 H, m), 4.61–4.60 (1 H, m), 3.41–3.38 (2 H, m), 2.82–2.80 (2 H, m), 2.58–2.55 (3 H, m), 2.31 (3 H, s), 2.15–2.08 (2 H, m), 1.82–1.67 (4 H, m), 1.54 (2 H, d, *J* = 13.11), 1.44–1.35 (2 H, m); ^13^C-NMR: 168.26, 145.65, 141.87, 136.90, 129.04, 128.29, 126.62, 125.78, 125.59, 78.74, 73.28, 52.47, 42.43, 38.27, 23.64, 23.05, 21.12; LC/MS-MS: 444.38 (M+H^+^).

*5-(4’-(Hydroxydiphenylmethyl)piperidin-1-yl)-1-(4-ethylphenyl)pentan-1-ol* (**3k**). Yield: 80 %; mp: 70 °C; IR: 2933, 1595, 1447, 1341, 1065; ^1^H-NMR (d_4_-MeOH): 8.46 (1 H, s), 7.49 (4 H, d, *J* = 8.27), 7.28–7.25 (4 H, m), 7.21 (2 H, d, *J* = 8.01), 7.16–7.12 (4 H, m), 4.56 (1 H, t, *J* = 6.90), 3.46 (2 H, d, *J* = 12.17), 2.99–2.90 (4 H, m), 2.82–2.78 (1 H, m), 2.58 (1 H, q, *J* = 7.58), 1.79–1.65 (8 H, m), 1.45–1.27 (2 H, m), 1.17 (3 H, t, *J* = 7.59); ^13^C-NMR (d_4_-MeOH): 145.74, 143.24, 142.06, 127.79, 127.40, 126.28, 125.69, 120.02, 78.43, 73.19, 52.47, 41.45, 37.99, 36.34, 28.15, 23.67, 22.65, 14.91; LC/MS-MS: 458.16 (M+H^+^).

*5-(4’-(Hydroxydiphenylmethyl)piperidin-1-yl)-1-(4-n-propylphenyl)pentan-1-ol* (**3l**). Yield: 64 %; mp: 83 °C; IR: 2933, 2362, 1594, 1447, 1342, 1242, 1066; ^1^H-NMR (d_4_-MeOH): 8.50 (1 H, s), 7.50–7.48 (4 H, m), 7.28–7.25 (4 H, m), 7.21–7.20 (2 H, m), 7.15–7.10 (4 H, m), 4.56–4.55 (1 H, m), 3.44 (2 H, d, *J* = 12.59), 2.94–2.87 (4 H, m), 2.81–2.76 (1 H, m), 2.54–2.52 (1 H, m), 1.75–1.58 (10 H, m), 1.44–1.27 (2 H, m), 0.91–0.88 (3 H, m); ^13^C-NMR (d_4_-MeOH): 147.02, 143.36, 142.79, 138.87, 129.47, 129.29, 129.03, 127.51, 127.38, 126.94, 126.86, 79.69, 74.46, 53.69, 42.78, 39.24, 38.57, 25.68, 25.32, 24.98, 23.93, 13.94; LC/MS-MS: 472.23 (M+H^+^).

*5-(4’-(Hydroxydiphenylmethyl)piperidin-1-yl)-1-(4-iso-propylphenyl)pentan-1-ol* (**3m**). Yield: 68 %; mp: 82 °C; IR: 3337, 2956, 2298, 1597, 1447, 1385, 1179; ^1^H-NMR: 8.40 (1 H, s), 7.49 (2 H, d, *J* = 7.54), 7.30–7.23 (6 H, m), 7.19–7.17 (4 H, m), 4.66–4.63 (1 H, m), 3.47–3.45 (2 H, m), 2.90–2.86 (3 H, m), 2.63–2.59 (3 H, m), 2.21–2.17 (2 H, m), 1.82–1.68 (4 H, m), 1.59 (2 H, d, *J* = 13.79). 1.51–1.41 (2 H, m), 1.23 (6 H, d, *J* = 6.92); ^13^C-NMR: 167.61, 148.08, 145.45, 142.05, 128.34, 126.71, 126.45, 125.82, 125.56, 78.82, 73.46, 42.43, 38.05, 33.79, 24.02, 23.55, 23.05; LC/MS-MS: 472.38 (M+H^+^).

*5-(4’-(Hydroxydiphenylmethyl)-piperidin-1-yl)-1-(4-n-butylphenyl)pentan-1-ol* (**3n**). Yield: 71 %; IR: 2930, 2362, 1592, 1448, 1343, 1066; ^1^H-NMR (d_4_-MeOH): 8.34 (1 H, s), 7.51 (4 H, d, *J* = 8.30), 7.31–7.27 (4 H, m), 7.23 (2 H, d, *J* = 8.04), 7.19–7.12 (4 H, m), 4.58 (1 H, t, *J* = 6.20), 3.49 (2 H, d, *J* = 12.36), 3.02–2.79 (5 H, m), 2.58 (2 H, t, *J* = 7.72), 1.83–1.66 (8 H, m), 1.60–1.48 (2 H, m), 1.31–1.29 (4 H, m), 0.92 (3 H, t, *J* = 7.32); ^13^C-NMR (d_4_-MeOH): 166.41, 145.71, 142.05, 141.77, 127.99, 127.80, 126.29, 125.69, 125.63, 78.43, 73.21, 52.43, 41.42, 37.96, 34.89, 33.65, 23.67, 22.64, 21.94, 12.89; LC/MS-MS: 486.20 (M+H^+^).

*5-(4’-(Hydroxydiphenylmethyl)-piperidin-1-yl)-1-(4-iso-butylphenyl)pentan-1-ol* (**3o**). Yield: 68 %; mp: 75 °C; IR: 2952, 2504, 1593, 1447, 1345, 1186, 1067; ^1^H-NMR (d_4_-MeOH): 8.36 (1 H, s), 7.53–7.50 (4 H, m), 7.31–7.27 (4 H, m), 7.24 (2 H, d, *J* = 8.02), 7.19–7.16 (2 H, m), 7.11 (2 H, d, *J* = 8.11), 4.59 (1 H, t, *J* = 5.99), 3.49 (2 H, d, *J* = 12.54), 3.03–2.80 (5 H, m), 2.44 (2 H, d, *J* = 7.18), 1.86–1.67 (9 H, m), 1.50–1.29 (2 H, m), 0.88 (6 H, d, *J* = 6.63); ^13^C-NMR (d_4_-MeOH): 147.12, 143.58, 141.96, 130.10, 129.20, 127.69, 127.10, 126.90, 79.84, 74.61, 53.88, 46.11, 42.82, 39.34, 31.51, 25.07, 24.06, 22.70; LC/MS-MS: 486.27 (M+H^+^).

*5-(4’-(Hydroxydiphenylmethyl)-piperidin-1-yl)-1-(4-tert-butylphenyl)pentan-1-ol* (**3p**). Yield: 58 %; mp: 88 °C; IR: 2950, 2359, 1597, 1447, 1345, 1033; ^1^H-NMR (d_4_-MeOH): 8.46 (1 H, s), 7.52–7.50 (4 H, m), 7.36 (2 H, d, *J* = 8.43), 7.31–7.25 (6 H, m), 7.19–7.16 (2 H, m), 4.60 (1 H, t, *J* = 6.84), 3.49 (2 H, d, *J* = 12.54), 3.02–2.94 (4 H, m), 2.86–2.80 (1 H, m), 1.84–1.67 (8 H, m), 1.49–1.32 (2 H, m), 1.29 (9 H, s); ^13^C-NMR (d_4_-MeOH): 153.94, 149.62, 145.67, 131.73, 130.23, 130.19, 129.60, 129.36, 129.33, 128.74, 82.38, 77.04, 56.43, 45.35, 41.83, 37.85, 34.37, 27.61, 26.54; LC/MS-MS: 486.27 (M+H^+^).

*4-(4’-(Hydroxydiphenylmethyl)piperidin-1-yl)-1-phenylbutan-1-ol* (**3q**). Yield: 76 %; mp: 94 °C; IR: 2778, 2361, 2339, 1595, 1448, 1356, 1064; ^1^H-NMR (d_4_-MeOH): 8.53 (1 H, s), 7.51 (4 H, d, *J* = 7.56), 7.36–7.23 (9 H, m), 7.17 (2 H, d, *J* = 7.29), 4.68–4.66 (1 H, m), 3.45–3.44 (2 H, m), 3.02–2.99 (2 H, m), 2.92–2.78 (3 H, m), 1.84–1.67 (8 H, m) [Lit. [14] 200 MHz, CDCl_3_: 7.67–7.11 (15 H, m), 4.60 (1 H, m), 3.43 (1 H, d, *J* = 7.00), 3.12 (1 H, d, *J* = 11.00), 2.94 (1 H, d, *J* = 11.00), 2.51–2.34 (5 H, m), 2.15–1.74 (4 H, m), 1.62–1.44 (4 H, m)]; ^13^C-NMR (d_4_-MeOH): 147.21, 145.98, 129.43, 129.17, 128.46, 127.65, 127.09, 126.91, 79.89, 74.27, 54.03, 53.91, 43.05, 37.21, 25.57, 22.08, 21.89; LC/MS-MS: 416.21 (M+H^+^).

*4-(4’-(Hydroxydiphenylmethyl)piperidin-1-yl)-1-(4-methylphenyl)butan-1-ol* (**3r**). Yield: 45 %; mp: 97 °C; IR: 2355, 1595, 1447, 1356, 1063; ^1^H-NMR (d_4_-MeOH): 8.54 (1 H, s), 7.52 (4 H, d, *J* = 7.90), 7.32–7.29 (4 H, m), 7.24 (2 H, d, *J* = 8.00), 7.20–7.14 (4 H, m), 4.65–4.63 (1 H, m), 3.48–3.45 (2 H, m), 3.04–3.00 (2 H, m), 2.94–2.79 (3 H, m), 2.31 (3 H, s), 1.83–1.68 (8 H, m); ^13^C-NMR (d_4_-MeOH): 170.06, 147.18, 142.87, 138.23, 130.02, 129.18, 127.66, 127.09, 126.89, 79.89, 74.14, 54.03, 53.91, 43.02, 37.13, 25.60, 22.08, 21.89, 21.12; LC/MS-MS: 430.14 (M+H^+^).

*4-(4’-(Hydroxydiphenylmethyl)piperidin-1-yl)-1-(4-ethylphenyl)butan-1-ol* (**3s**). Yield: 41 %; mp: 99 °C; IR: 2959, 1596, 1490, 1447, 1344, 1066; ^1^H-NMR (d_4_-MeOH): 8.55 (1 H, s), 7.52 (4 H, d, *J* = 7.83), 7.31–7.25 (6 H, m), 7.19–7.17 (4 H, m), 4.65–4.63 (1 H, m), 3.41–3.36 (2 H, m), 2.95–2.91 (2 H, m), 2.79–2.76 (3 H, m), 2.65–2.60 (2 H, m), 1.82–1.66 (8 H, m), 1.21 (3 H, t, *J* = 7.61); ^13^C-NMR (d_4_-MeOH): 170.26, 147.31, 144.78, 143.23, 143.21, 129.17, 128.88, 127.63, 127.13, 127.01, 79.97, 74.28, 74.17, 58.31, 54.21, 54.08, 43.36, 37.38, 29.56, 25.82, 22.39, 22.19, 16.29; LC/MS-MS: 444.28 (M+H^+^).

*4-(4’-(Hydroxydiphenylmethyl)piperidin-1-yl)-1-(4-n-propylphenyl)butan-1-ol* (**3t**). Yield: 76 %; mp: 104 °C; IR: 2933, 2363, 1593, 1491, 1447, 1342, 1065; ^1^H-NMR (d_4_-MeOH): 8.56 (1 H, s), 7.52 (4 H, d, *J* = 7.74), 7.31–7.25 (6 H, m), 7.19–7.15 (4 H, m), 4.64–4.63 (1 H, m), 3.38–3.32 (2 H, m), 2.90–2.86 (2 H, m), 2.78–2.70 (3 H, m), 2.59–2.56 (2 H, m), 1.78–1.60 (8 H, m), 0.93 (3 H, t, *J* = 7.35); ^13^C-NMR (d_4_-MeOH): 147.35, 143.27, 143.03, 129.49, 129.12, 127.57, 127.11, 126.91, 79.98, 74.20, 58.38, 54.26, 54.12, 43.51, 38.71, 25.90, 25.79, 22.31, 14.06; LC/MS-MS: 460.23 (M+H^+^).

*4-(4’-(Hydroxydiphenylmethyl)piperidin-1-yl)-1-(4-iso-propylphenyl)butan-1-ol* (**3u**). Yield: 69 %; mp: 173 °C; IR: 2959, 2363, 1606, 1446, 1327, 1176, 1071; ^1^H-NMR (d_4_-MeOH): 8.52 (1 H, s), 7.51 (4 H, d, *J* = 7.62), 7.31–7.26 (6 H, m), 7.21–7.16 (4 H, m), 4.65–4.63 (1 H, m), 3.47–3.45 (2 H, m), 3.04–3.01 (2 H, m), 2.94–2.78 (4 H, m), 1.83–1.68 (8 H, m), 1.22 (6 H, t, *J* = 6.93); ^13^C-NMR (d_4_-MeOH): 149.42, 147.18, 143.27, 129.18, 127.67, 127.41, 127.08, 126.97, 79.86, 74.13, 53.97, 53.87, 49.01, 42.97, 37.06, 35.10, 25.52, 24.46, 22.01; LC/MS-MS: 458.13 (M+H^+^).

*4-(4’-(Hydroxydiphenylmethyl)piperidin-1-yl)-1-(4-n-butylphenyl)butan-1-ol* (**3v**). Yield: 65 %; mp: 175 °C; IR: 2932, 2366, 1596, 1448, 1342, 1065; ^1^H-NMR (d_4_-MeOH): 8.54 (1 H, s), 7.51 (4 H, d, *J* = 7.48), 7.30–7.24 (6 H, m), 7.18–7.14 (4 H, m), 4.64–4.62 (1 H, m), 3.41–3.35 (2 H, m), 2.93–2.90 (2 H, m), 2.80–2.74 (3 H, m), 2.60–2.57 (2 H, m), 1.81–1.65 (8 H, m), 1.60–1.54 (2 H, m), 1.37–1.32 (2 H, m), 0.92 (3 H, t, *J* = 7.40); ^13^C-NMR (d_4_-MeOH): 147.27, 143.29, 143.18, 129.45, 129.14, 127.61, 127.10, 126.92, 85.09, 79.96, 74.27, 54.08, 43.34, 37.33, 36.27, 35.01, 31.79, 25.81, 23.31, 22.37, 19.23, 14.27; LC/MS-MS: 472.20 (M+H^+^).

*4-(4’-(Hydroxydiphenylmethyl)piperidin-1-yl)-1-(4-iso-butylphenyl)butan-1-ol* (**3w**). Yield: 68 %; mp: 177 °C; IR: 2950, 2361, 1595, 1447, 1328, 1171, 1067; ^1^H-NMR (d_4_-MeOH): 8.54 (1 H, s), 7.52–7,50 (4 H, d, *J* = 7.42), 7.30–7.24 (6 H, m), 7.18–7.15 (2 H, m), 7.13–7.11 (2 H, m), 4.65–4.62 (1 H, m), 3.42–3.38 (2 H, m), 2.96–2.93 (2 H, m), 2.83–2.74 (3 H, m), 2.45 (2 H, d, *J* = 7.18), 1.86–1.66 (9 H, m), 0.88 (6 H, d, *J* = 6.63); ^13^C-NMR (d_4_-MeOH): 147.29, 143.32, 142.10, 130.18, 129.17, 127.64, 127.12, 126.81, 79.95, 74.27, 58.25, 54.15, 46.12, 43.29, 37.29, 31.52, 25.76, 22.71, 22.32; LC/MS-MS: 472.34 (M+H^+^).

*4-tert-Butylphenyl-6-bromohexanoate* (**4a**). Yield: 61 %; ^1^H-NMR: 7.41–7.40 (2 H, d, *J* = 8.59), 7.03–7.02 (2 H, d, *J* = 8.77), 3.47–3.44 (2 H, t, *J* = 6.72), 2.61–2.58 (2 H, t, *J* = 7.38), 1.98–1.92 (2 H, m), 1.84–1.78 (2 H, m), 1.63–1.59 (2 H, m), 1.34 (9 H, s); ^13^C-NMR: 172.06, 148.59, 148.36, 126.31, 120.84, 77.33, 77.06, 76.82, 34.49, 34.17, 33.43, 32.41, 31.44, 27.64, 24.12.

N-(4-tert-Butylphenyl)-6-bromohexanamide (**4b**). Yield: 71 %; ^1^H-NMR: 7.43–7.41 (2 H, d, *J* = 8.59), 7.34–7.32 (2 H, d, *J* = 8.58), 3.43–3.40 (2 H, t, *J* = 6.70), 2.38–2.25 (2 H, t, *J* = 7.38), 1.93–1.87 (2 H, m), 1.79–1.73 (2 H, m), 1.56–1.50 (2 H, m), 1.30 (9 H, s); ^13^C-NMR: 170.95, 147.37, 135.09, 125.80, 119.72, 38.74, 37.37, 33.53, 32.43, 31.34, 27.72, 24.70.

*4-tert-Butylphenyl-6-(4’-(hydroxydiphenylmethyl)pi-peridin-1-yl)hexanoate* (**5a**). Yield: 52 %; IR: 3398, 2962, 2359, 1749, 1591, 1509, 1448, 1347, 1207, 1172, 1147, 1108, 1017, 951, 839, 749, 702; ^1^H-NMR: 8.46 (1 H, s), 7.54–7.53 (4 H, m), 7.41–7.39 (2 H, m), 7.30–7.29 (4 H, m), 7.18–7.17 (2 H, m), 6.99–6.98 (2 H, m), 3.50 (2 H, s), 3.04–2.82 (5 H, m), 2.60–2.59 (2 H, m), 1.83–1.70 (8 H, m), 1.47 (2 H, s), 1.30 (9 H, s); ^13^C-NMR: 173.82, 169.06, 169.02, 149.95, 147.27, 129.25, 127.76, 127.36, 127.12, 122.12, 79.88, 53.77, 42.93, 35.38, 34.68, 31.94, 27.74, 27.14, 25.35, 24.85, 24.41, 24.03, 23.82; LC/MS-MS: 514.25 (M+H^+^). 

*N-(4-tert-Butylphenyl)-6-(4-(hydroxydiphenylmethyl)piperidin-1-yl)hexanamide* (**5b**). Yield: 36 %; IR: 3286, 2959, 1662, 1598, 1537, 1448, 1346, 1111, 1031, 953, 839, 749, 702; ^1^H-NMR: 8.44 (1 H, s), 7.53–7.52 (4 H, d, *J* = 7.36), 7.46–7.44 (2 H, d, *J* = 8.70), 7.34–7.28 (6 H, m), 7.19–7.16 (2 H, t, *J* = 7.35), 3.63 (1 H, s), 3.53–3.51 (2 H, m), 3.07–2.99 (4 H, m), 2.87–2.81 (1 H, m), 2.41–2.38 (2 H, t, *J* = 7.16), 1.78–1.71 (8 H, m), 1.46–1.43 (2 H, m), 1.30 (9 H, s); ^13^C-NMR: 174.07, 148.32, 147.13, 129.20, 127.69, 127.11, 126.58, 121.09, 42.88, 42.85, 37.31, 35.25, 31.82, 27.22, 27.18, 26.06, 26.02, 24.90; LC/MS-MS: 513.27 (M+H^+^).

*5-Bromo-1-(4-tert-butylphenyl)hexan-1-ol* (**6a**). Yield: 74 %; ^1^H-NMR (d_4_-MeOH): 7.26–7.24 (2 H, d, *J* = 7.85), 7.13–7.12 (2 H, d, *J* = 7.81), 4.47–4.44 (1 H, t, *J* = 6.80), 3.27–3.24 (2 H, t, *J* = 6.72), 2.43–2.39 (1 H, m), 1.74–1.56 (4 H, m), 1.34–1.33 (4 H, m), 1.22 (9 H, s); ^13^C-NMR: 150.11, 141.63, 125.41, 125.08, 73.89, 38.49, 34.31, 33.64, 32.54, 31.26, 27.90, 24.86.

*1-(5-Bromohexyl)-4-tert-butylbenzene* (**7a**).

To a mixture of indium(III) chloride (33 mg) and **6a** (920 mg) in dry dichloromethane chlorodiphenylsilane (1.2 mL) was added dropwise. After stirring at room temperature for 3 hours the solution was hydrolyzed with a little amount of water. Diethyl ether was added, the organic layer separated and the water phase extracted with diethyl ether two times. The combined organic layers were dried, the solvent removed under reduced pressure and the raw product was purified by column chromatography (ethyl acetate/n-hexane: 1/12, followed by pure hexane). Yield: 23 %; ^1^H-NMR (d_4_-MeOH): 7.26–7.24 (2 H, d, *J* = 8.27), 7.06–7.05 (2 H, d, *J* = 8.26), 3.34–3.31 (2 H, t, *J* = 6.83), 2.55–2.52 (2 H, t, *J* = 7.88), 1.82–1.78 (2 H, m), 1.59–1.55 (2 H, m), 1.43–1.38 (2 H, m), 1.34–1.30 (2 H, m), 1.27 (9 H, s); ^13^C-NMR: 148.33, 139.38, 127.97, 125.08, 35.23, 34.28, 33.78, 32.73, 31.41, 31.18, 28.45, 28.02.

*(1-(6-(4-tert-Butylphenyl)hexyl)piperidin-4-yl)diphenylmethanol* (**8a**). Yield: 40 %; IR: 2933, 2363, 1662, 1596, 1491, 1447, 1343, 1172, 1067, 955, 832, 749, 702; ^1^H-NMR: 7.46–7.45 (4 H, d, *J* = 8.49), 7.26–7.22 (6 H, t, *J* = 8.45), 7.14–7.11 (2 H, t, *J* = 7.31), 7.05–7.03 (2 H, d, *J* = 8.26), 3.43–3.41 (2 H, d, *J* = 11.05), 2.81–2.77 (2 H, m), 2.58–2.55 (2 H, m), 2.52–2.49 (2 H, t, *J* = 7.85), 2.15–2.13 (2 H, m), 1.68–1.67 (2 H, m), 1.58–1.55 (4 H, m), 1.31–1.29 (4 H, m), 1.27 (9 H, s); ^13^C-NMR: 148.45, 145.48, 139.22, 128.27, 127.96, 126.65, 125.54, 125.12, 78.78, 52.52, 52.5, 52.46, 52.45, 45.38, 42.50, 35.13, 34.30, 31.39, 31.09, 28.72, 26.79, 23.76, 23.31, 23.27; LC/MS-MS: 485.00 (M+H^+^).

*1-(4-iso-Butylphenyl)-5-(piperidin-1-yl)pentan-1-one* (**9a**). Yield: 78 %; mp: 143 °C; IR: 2955, 2869, 1739, 1676, 1604, 1453, 1368, 1273, 1184, 1022; ^1^H-NMR: 7.93–7.91 (2 H, d, *J* = 8.31), 7.30–7.28 (2 H, d, *J* = 8.25), 3.13–3.07 (4 H, m), 2.56–2.55 (2 H, d, *J* = 7.23), 1.95–1.68 (12 H, m), 1.35–1.32 (1 H, m), 0.92–0.91 (6 H, d, *J* = 6.63); ^13^C-NMR: 201.45, 149.18, 136.01, 130.53, 129.21, 64.32, 64.29, 58.04, 54.22, 52.98, 46.31, 38.41, 31.35, 24.60, 24.27, 22.82, 22.69, 22.15, 16.65, 16.60; LC/MS-MS: 302.93 (M+H^+^).

*1-(4-iso-Butylphenyl)-5-(4-methylpiperidin-1-yl)pentan-1-one* (**9b**). Yield: 21 %; IR: 2956, 2361, 1679, 1605, 1457, 1414, 1369, 1336, 1182, 977, 855, 759; ^1^H-NMR: 7.88–7.86 (2 H, d, *J* = 8.24), 7.26–7.24 (2 H, d, *J* = 8.19), 3.52–3.51 (2 H, m), 3.06–3.03 (2 H, t, *J* = 6.84), 2.99–2.96 (2 H, m), 2.64–2.54 (4 H, m), 1.93–1.75 (9 H, m), 1.63–1.62 (1 H, m), 1.04–1.02 (3 H, d, *J* = 6.37), 0.93–0.92 (6 H, d, *J* = 6.62); ^13^C-NMR: 199.10, 147.76, 134.45, 129.31, 127.96, 45.36, 37.37, 30.07, 23.41, 22.31, 21.19; LC/MS-MS: 315.94 (M+H^+^).

*1-(4-iso-Butylphenyl)-5-(4-hydroxypiperidin-1-yl)-pentan-1-one* (**9c**). Yield: 68 %; IR: 3345, 2954, 2495, 1735, 1679, 1605, 1465, 1414, 1372, 1239, 1181, 1042, 978, 852, 763; ^1^H-NMR: 7.90–7.88 (2 H, d, *J* = 8.27), 7.26–7.25 (2 H, d, *J* = 8.20), 3.93 (1 H, s), 3.38 (2 H, s), 3.19–3.05 (5 H, m), 2.52–2.51 (2 H, d, *J* = 7.21), 2.06–2.03 (2 H, m), 1.89–1.73 (8 H, m), 0.88–0.87 (6 H, d, *J* = 6.63); ^13^C-NMR: 201.44, 149.14, 136.02, 130.54, 129.23, 71.13, 57.71, 46.32, 38.47, 31.83, 31.35, 24.77, 22.72, 22.13, 20.94; LC/MS-MS: 318.11 (M+H^+^).

*5-(4-Benzylpiperidin-1-yl)-1-(4-iso-butylphenyl) pentan-1-one* (**9d**). Yield: 34 %; mp: 143 °C; IR: 3454, 2931, 1682, 1605, 1455, 1241, 1112; ^1^H-NMR: 7.86–7.84 (2 H, d, *J* = 8.26), 7.27–7.10 (7 H, m), 7.95–7.92 (2 H, m), 2.86 (2 H, d, *J* = 11.65), 2.51–2.50 (4 H, d, *J* = 7.21), 2.33–2.30 (2 H, m), 1.90–1.80 (3 H, m), 1.80–1.69 (2 H, m), 1.61–1.46 (5 H, m), 1.32–1.20 (2 H, m), 0.89–0.88 (6 H, d, *J* = 6.62); ^13^C-NMR: 199.89, 147.24, 140.66, 134.76, 129.18, 129.03, 128.05, 127.98, 125.66, 77.01, 70.54, 58.67, 53.90, 45.30, 43.18, 38.25, 37.91, 32.15, 30.02, 26.69, 22.52, 22.27; LC/MS-MS: 392.07 (M+H^+^).

*1-(4-iso-Butylphenyl)-5-(piperidin-1-yl)pentan-1-ol* (**10a**). Yield: 94 %; mp: 64 °C; IR: 3386, 2952, 1587, 1446, 1347, 1018; ^1^H-NMR (d_4_-MeOH): 8.55 (1 H, s), 7.25–7.24 (2 H, d, *J* = 8.01), 7.12–7.10 (2 H, d, *J* = 8.36), 4.60–4.58 (1 H, m), 3.31–2.98 (3 H, m), 2.85–2.82 (2 H, m), 2.46–2.45 (2 H, d, *J* = 7.18), 1.87–1.61 (10 H, m), 1.48–1.28 (3 H, m), 0.90–0.88 (2 H, d, *J* = 6.62); ^13^C-NMR (d_4_-MeOH): 170.38, 143.64, 141.89, 130.07, 126.92, 74.61, 58.69, 54.46, 46.13, 31.50, 25.46, 25.43, 24.26, 24.15, 23.40, 22.70; LC/MS-MS: 306.92 (M+H^+^).

*1-(4-iso-Butylphenyl)-5-(4-methylpiperidin-1-yl)pentan-1-ol* (**10b**). Yield: 57 %; IR: 3349, 2953, 2667, 1717, 1594, 1458, 1177, 1051, 948, 848; ^1^H-NMR (d_4_-MeOH): 7.23–7.21 (2 H, d, *J* = 8.01), 7.11–7.09 (2 H, d, *J* = 7.82), 3.54–3.53 (2 H, m), 2.92–2.90 (2 H, m), 2.56–2.54 (2 H, m), 2.45–2.44 (2 H, d, *J* = 7.15), 1.87–1.70 (10 H, m), 1.50–1.39 (3 H, m), 1.02–1.01 (3 H, d, *J* = 6.47), 0.89–0.88 (6 H, d, *J* = 6.61); ^13^C-NMR: 141.75, 141.05, 129.21, 125.55, 73.65, 57.35, 52.99, 45.08, 37.98, 30.19, 23.04, 22.36, 20.97; LC/MS-MS: 318.04 (M+H^+^).

*1-(4-iso-Butylphenyl)-5-(4-hydroxypiperidin-1-yl)-pentan-1-ol* (**10c**). Yield: 47 %; IR: 3357, 2950, 2868, 2670, 1466, 1383, 1040, 973, 849, 802, 609; ^1^H-NMR: 7.28–7.27 (2 H, d, *J* = 8.01), 7.14–7.13 (2 H, d, *J* = 8.01), 4.64–4.62 (1 H, t, *J* = 7.19), 4.10–3.56 (2 H, m), 3.37 (1 H, s), 3.25 (1 H, s), 3.09–3.06 (2 H, t, *J* = 8.17), 2.48–2.47 (2 H, d, *J* = 7.17), 2.12–1.77 (10 H, m), 1.52–1.38 (2 H, m), 0.91–0.90 (6 H, d, *J* = 6.62); ^13^C-NMR: 143.61, 141.94, 130.10, 126.93, 74.62, 52.21, 46.13, 39.39, 31.51, 30.98, 24.07, 22.72; LC/MS-MS: 319.93 (M+H^+^).

*5-(4-Benzylpiperidin-1-yl)-1-(4-iso-butylphenyl) pentan-1-ol* (**10d**). Yield: 45 %; mp: 64 °C; IR: 2929, 2361, 1454, 1275; ^1^H-NMR (d_4_-MeOH): 7.30–7.17 (7 H, m), 7.12–7.10 (2 H, d, *J* = 7.99), 7.19–7.12 (4 H, m), 4.61–4.59 (1 H, m), 3.50–3.49 (2 H, m), 3.03–2.87 (4 H, m), 2.62–2.61 (2 H, d, *J* = 7.17), 2.62–2.61 (2 H, m), 1.89–1.71 (8 H, m), 1.52–1.47 (4 H, m), 0.89–0.88 (2 H, d, *J* = 6.05); ^13^C-NMR (d_4_-MeOH): 143.59, 141.95, 140.50, 130.19, 130.10, 129.50, 127.40, 126.91, 74.61, 46.12, 39.36, 31.51, 25.10, 24.07, 22.71; LC/MS-MS: 394.24 (M+H^+^).

*1-(4-iso-Butylphenyl)-5-morpholinopentan-1-one* (**11a**). Yield: 63 %; IR: 2867, 1681, 1606, 1466, 1353, 1251, 1182, 1114, 988, 949, 861, 734; ^1^H-NMR: 7.85–7.83 (2 H, d, *J* = 8.21), 7.22–7.20 (2 H, d, *J* = 8.14), 3.88–3.86 (4 H, t, *J* = 4.76), 3.00–2.98 (2 H, t, *J* = 6.22), 2.90 (4 H, s), 2.79–2.76 (2 H, t, *J* = 7.28), 2.52–2.50 (2 H, d, *J* = 7.19), 1.89–1.87 (1 H, m), 1.76–1.75 (4 H, m), 0.89–0.88 (6 H, d, *J* = 6.62); ^13^C-NMR: 199.68, 147.25, 134.66, 129.14, 127.88, 70.57, 66.73, 58.56, 53.58, 45.24, 38.06, 29.96, 25.97, 22.02, 22.14; LC/MS-MS: 304.12 (M+H^+^).

*1-(4-iso-Butylphenyl)-5-morpholinopentan-1-ol* (**12a**). Yield: 26 %; IR: 3422, 2952, 2868, 2465, 1722, 1599, 1514, 1466, 1366, 1264, 1105, 977, 849, 803, 620 ^1^H-NMR: 7.26–7.24 (2 H, d, *J* = 8.00), 7.13–7.11 (2 H, d, *J* = 8.03), 4.62–4.60 (1 H, m), 3.89 (4 H, s), 3.20 (4 H, s), 3.06–3.03 (2 H, t, *J* = 8.31), 2.47–2.45 (2 H, d, *J* = 7.18), 1.84–1.74 (5 H, m), 1.49–1.35 (2 H, m), 0.90–0.88 (6 H, d, *J* = 6.63) ^13^C-NMR: 143.62, 141.94, 130.10, 126.92, 74.63, 71.51, 65.28, 58.56, 53.25, 46.13, 45.60, 39.43, 31.51, 24.81, 24.00, 22.71 LC/MS-MS: 306.08 (M+H^+^).

*1-(5-(4-iso-Butylphenyl)-5-oxopentyl)piperidin-4-one* (**13a**). Yield: 3 %; IR: 2955, 2869, 2808, 1716, 1678, 1606, 1570, 1466, 1307, 1275, 1183, 954, 846, 796; ^1^H-NMR: 7.93–7.90 (2 H, m), 7.30–7.28 (2 H, m), 2.79–2.76 (2 H, t, *J* = 6.16), 2.56–2.42 (9 H, m), 1.93–1.90 (1 H, m), 1.78–1.60 (7 H, m), 0.92–0.91 (6 H, d, *J* = 6.63); ^13^C-NMR: 199.42, 129.34, 127.99, 70.48, 56.68, 52.04, 45.41, 39.37, 37.67, 30.04, 22.27, 21.65; LC/MS-MS: 316.15 (M+H^+^).

*5-(3-(Hydroxydiphenylmethyl)pyrrolidin-1-yl)-1-(4-iso-butylphenyl)pentan-1-one* (**15a**). Yield: 36 %; IR: 2957, 1679, 1605, 1448, 1367, 1182, 984, 751, 700; ^1^H-NMR: 7.79–7.77 (2 H, d, *J* = 8.15), 7.43–7.40 (4 H, t, *J* = 8.27), 7.27–7.22 (4 H, m), 7.18–7.14 (4 H, m), 3.75–3.72 (1 H, m), 3.07–2.92 (8 H, m), 2.49–2.47 (2 H, d, *J* = 7.19), 2.16–1.99 (2 H, m), 1.86–1.84 (1 H, m), 1.72–1.69 (4 H, m), 0.87–0.85 (6 H, d, *J* = 6.62); ^13^C-NMR: 199.11, 147.86, 145.41, 134.32, 129.37, 128.65, 128.46, 127.97, 127.91, 127.04, 125.51, 125.45, 78.48, 55.24, 54.54, 45.61, 45.36, 37.28, 30.07, 25.56, 25.13, 22.30, 20.89; LC/MS-MS: 470.10 (M+H^+^).

*5-(3-(Hydroxydiphenylmethyl)pyrrolidin-1-yl)-1-(4-iso-butylphenyl)pentan-1-ol* (**16a**). Yield: 48 %; IR: 3351, 2954, 2373, 1597, 1448, 1338, 1176, 1107, 1067, 1034, 850, 750, 703; ^1^H-NMR: 7.45–7.42 (4 H, t, *J* = 7.67), 7.29–7.24 (4 H, m), 7.19–7.16 (4 H, m), 7.08–7.06 (2 H, d, *J* = 7.97), 4.62–4.59 (1 H, m), 3.77–3.74 (1 H, m), 2.91–2.88 (6 H, m), 2.43–2.42 (2 H, d, *J* = 7.14), 2.19–2.15 (2 H, m), 1.83–1.79 (1 H, m), 1.76–1.64 (4 H, m), 1.46–1.35 (2 H, m), 0.87–0.86 (6 H, d, *J* = 6.61); ^13^C-NMR: 145.66, 141.83, 141.02, 129.20, 128.63, 128.47, 127.19, 127.07, 125.58, 125.52, 125.49, 78.49, 73.66, 61.84, 54.56, 54.38, 45.09, 37.95, 30.20, 25.63, 25.32, 22.91, 22.37; LC/MS-MS: 472.20 (M+H^+^).
